# Interdisciplinary perspectives on computed tomography in sepsis: survey among medical doctors at a large university medical center

**DOI:** 10.1007/s00330-023-09842-3

**Published:** 2023-07-14

**Authors:** Maria Isabel Opper Hernando, Denis Witham, Peter Richard Steinhagen, Stefan Angermair, Wolfgang Bauer, Friederike Compton, Andreas Edel, Jan Kruse, York Kühnle, Gunnar Lachmann, Susanne Marz, Holger Müller-Redetzky, Jens Nee, Oliver Paul, Damaris Praeger, Carsten Skurk, Miriam Stegemann, Alexander Uhrig, Stefan Wolf, Elke Zimmermann, Kerstin Rubarth, Myrto Bolanaki, Joachim Seybold, Marc Dewey, Julian Pohlan

**Affiliations:** 1 Department of Radiology, Charité – Universitätsmedizin Berlin, corporate member of Freie Universität Berlin and Humboldt-Universität zu Berlin, Charitéplatz 1, 10117 Berlin, Germany; 2 Department of Cardiology, Charité – Universitätsmedizin Berlin, corporate member of Freie Universität Berlin and Humboldt-Universität zu Berlin, Charitéplatz 1, 10117 Berlin, Germany; 3 Berlin Institute of Health at Charité – Universitätsmedizin Berlin, Charitéplatz 1, 10117 Berlin, Germany; 4 Department of Gastroenterology and Hepatology, Charité – Universitätsmedizin Berlin, corporate member of Freie Universität Berlin and Humboldt-Universität zu Berlin, Charitéplatz 1, 10117 Berlin, Germany; 5 Department of Anesthesiology and Operative Intensive Care Medicine, Charité - Universitätsmedizin Berlin, corporate member of Freie Universität Berlin and Humboldt-Universität zu Berlin, Hindenburgdamm 30, 12203 Berlin, Germany; 6 Emergency Department, Charité - Universitätsmedizin Berlin, corporate member of Freie Universität Berlin and Humboldt-Universität zu Berlin, Hindenburgdamm 30, 12203 Berlin, Germany; 7 Medical Clinic with focus on Nephrology and Internal Intensive Care Medicine, Charité - Universitätsmedizin Berlin, corporate member of Freie Universität Berlin and Humboldt-Universität zu Berlin, Hindenburgdamm 30, 12203 Berlin, Germany; 8 Department of Anesthesiology and Operative Intensive Care Medicine, Charité – Universitätsmedizin Berlin, corporate member of Freie Universität Berlin and Humboldt-Universität zu Berlin, Charitéplatz 1, 10117 Berlin, and Augustenburger Platz 1, 13353 Berlin, Germany; 9 Medical Clinic with focus on Nephrology and Internal Intensive Care Medicine, Charité – Universitätsmedizin Berlin, corporate member of Freie Universität Berlin and Humboldt-Universität zu Berlin, Augustenburger Platz 1, 13353 Berlin, Germany; 10 Department of Cardiology, Angiology and Intensive Care Medicine, Charité – Universitätsmedizin Berlin, corporate member of Freie Universität Berlin and Humboldt-Universität zu Berlin, Augustenburger Platz 1, 13353 Berlin, Germany; 11 Surgical Clinic – Interdisciplinary Anesthesiological and Surgical Intensive Care Unit, Charité – Universitätsmedizin Berlin, corporate member of Freie Universität Berlin and Humboldt-Universität zu Berlin, Charitéplatz 1, 10117 Berlin, and Augustenburger Platz 1, 13353 Berlin, Germany; 12 Department of Infectious Diseases, Pneumology and Intensive Care Medicine Group, Charité – Universitätsmedizin Berlin, corporate member of Freie Universität Berlin and Humboldt-Universität zu Berlin, Charitéplatz 1, 10117 Berlin, Germany; 13 Department of Cardiology, Angiology and Intensive Care Medicine, Charité – Universitätsmedizin Berlin, corporate member of Freie Universität Berlin and Humboldt-Universität zu Berlin, Charitéplatz 1, 10117 Berlin, Germany; 14 Department of Cardiology, Angiology and Intensive Care Medicine, Charité - Universitätsmedizin Berlin, corporate member of Freie Universität Berlin and Humboldt-Universität zu Berlin, Hindenburgdamm 30, 12203 Berlin, Germany; 15 Department of Infectious Diseases, Pneumology and Intensive Care Medicine Group, Charité – Universitätsmedizin Berlin, corporate member of Freie Universität Berlin and Humboldt-Universität zu Berlin, Augustenburger Platz 1, 13353 Berlin, Germany; 16 Department of Neurosurgery with Pediatric Neurosurgery Unit, Charité – Universitätsmedizin Berlin, corporate member of Freie Universität Berlin and Humboldt-Universität zu Berlin, Charitéplatz 1, 10117 Berlin, Germany; 17 Institute of Biometry and Clinical Epidemiology, Charité – Universitätsmedizin Berlin, corporate member of Freie Universität Berlin and Humboldt-Universität zu Berlin, Charitéplatz 1, 10117 Berlin, Germany; 18 Institute of Medical Informatics, Charité – Universitätsmedizin Berlin, corporate member of Freie Universität Berlin and Humboldt-Universität zu Berlin, Charitéplatz 1, 10117 Berlin, Germany; 19 Emergency Department, Charité – Universitätsmedizin Berlin, corporate member of Freie Universität Berlin and Humboldt-Universität zu Berlin, Charitéplatz 1, 10117 Berlin, and Augustenburger Platz 1, 13353 Berlin, Germany; 20 Office for Intercultural Competencies in the Berlin Health Care System, Charité – Universitätsmedizin Berlin, corporate member of Freie Universität Berlin and Humboldt-Universität zu Berlin, Charitéplatz 1, 10117 Berlin, Germany

**Keywords:** Tomography, X-ray computed, Sepsis, Surveys and questionnaires, Physicians, Clinical reasoning

## Abstract

**Objectives:**

This study aims to describe physicians’ perspectives on the use of computed tomography (CT) in patients with sepsis.

**Methods:**

In January 2022, physicians of a large European university medical center were surveyed using a web-based questionnaire asking about their views on the role of CT in sepsis. A total of 371 questionnaires met the inclusion criteria and were analyzed using work experience, workplace, and medical specialty of physicians as variables. Chi-square tests were performed.

**Results:**

Physicians considered the ability to detect an unknown focus as the greatest benefit of CT scans in sepsis (70.9%, *n *= 263/371). Two clinical criteria — “signs of decreased vigilance” (89.2%, *n *= 331/371) and “increased catecholamine demand” (84.7%, *n *= 314/371) — were considered highly relevant for a CT request. Elevated procalcitonin (82.7%, *n *= 307/371) and lactate levels (83.6%, *n *= 310/371) were consistently found to be critical laboratory values to request a CT. As long as there is evidence of infection in one organ region, most physicians (42.6%, *n *= 158/371) would order a CT scan based on clinical assessment. Combined examination of the chest, abdomen, and pelvis was favored (34.8%, *n *= 129/371) in cases without clinical clues of an infection source. A time window of ≥ 1–6 h was preferred for both CT examinations (53.9%, *n *= 200/371) and CT-guided interventions (59.3%, *n *= 220/371) in patients with sepsis.

**Conclusion:**

Despite much consensus, there are significant differences in attitudes towards the use of CT in septic patients among physicians from different workplaces and medical specialties. Knowledge of these perspectives may improve patient management and interprofessional communication.

**Key Points:**

- *Despite interdisciplinary consensus on the use of CT in sepsis, statistically significant differences in the responses are apparent among physicians from different workplaces and medical specialties.*
- *The detection of a previously unknown source of infection and the ability to plan interventions and/or surgery based on CT findings are considered key advantages of CT in septic patients.*
- *Timing of CT reflects the requirements of specific disciplines.*

**Supplementary information:**

The online version contains supplementary material available at 10.1007/s00330-023-09842-3.

## Introduction

Sepsis is defined as a life-threatening organ dysfunction caused by a dysregulated host response to infection [1]. Early detection and prompt initiation of appropriate treatment within the first hours after the onset of sepsis may improve outcomes [2, 3]. In addition to rapidly establishing the diagnosis of sepsis and administrating antibiotics as part of sepsis bundles, timely identification of the source of infection is recommended [2, 4]. A targeted search should be performed to enable early source control through adequate antibiotic treatment, surgery, and intervention [2, 3]. Imaging is recommended to confirm or rule out a suspected focus [3, 5]. However, imaging modalities are not addressed in the Surviving Sepsis Campaign (SSC) from 2021 or prior guidelines [2, 6, 7]. Furthermore, the guidelines do not provide recommendations for the optimal time window, body regions, or established clinical parameters that indicate imaging. Therefore, the SSC addressed the need for future research to determine the optimal time window and method of imaging most beneficial in patients with sepsis [2].

Along with chest radiography (CXR) and ultrasonography (USG), computed tomography (CT) is considered one of the imaging modalities of choice in patients with an unclear focus of infection [8]. It is, therefore, frequently used in clinical practice. CT allows full-body scans with high spatial resolution in a short period [9]. Imaging examinations are needed to localize a focal area, assess organ conditions, and identify possible complications [10]. In patients with sepsis, the chest, abdomen, and pelvis are the most commonly imaged organ regions [11]. In a previous study of our group, the most common location of infectious foci identified by CT was the chest, followed by the abdomen and genitourinary tract across different hospital settings [12–14]. As CT provides an accurate visualization of the thoracic and abdominal cavity and related pathologies [9], it is an appropriate tool for patients with suspected sepsis. Furthermore, the results of a CT scan have been shown to influence the decision-making process in an emergency care setting [15].

This survey was conducted to obtain an overview of physicians’ perspectives on the use of CT in patients with sepsis.

## Methods

### Setting

This study was conducted at a large European university hospital. All physicians of the hospital were contacted and thus given the opportunity to participate in our survey. The second part of this analysis will be published separately.

### Survey

The interdisciplinary team of authors developed and consented to a questionnaire on the role of CT scans in patients with sepsis. The survey was recently validated in a group of final-year medical students (Pohlan J, Opper Hernando MI, et al. Final-year medical students’ perspective: a survey on the use of computed tomography in sepsis; in review) with iterative changes to its structure. The local ethics committee approved the study (reference number EA1/203/21). The staff council authorized the survey, which was conducted in accordance with the Declaration of Helsinki.

### Questionnaire structure

The questionnaire was divided into several sections. First, physicians had to agree to the data protection declaration and indicate whether they had already participated in the survey. Demographic data requested included position (assistant, board-certified, and senior or chief physicians), work experience since licensure in years, medical specialty, possible sepsis-related fellowships, and workplace (> 50% of working time). Assistant physicians are defined as licensed physicians who are in residency training to become board-certified physicians. In addition, participants were asked whether they were involved in the management of septic patients in their daily clinical practice.

Indicating their responses on a 4-point Likert scale {(1) strongly disagree, (2) somewhat disagree, (3) somewhat agree, and (4) strongly agree}, physicians were first asked whether they felt the listed options represented a major benefit of CT scans in septic patients. Next, physicians had to indicate whether they agreed or disagreed with the listed clinical and ancillary criteria in support of a CT scan or of particular relevance to the indication for a CT examination. Furthermore, participants were asked to prioritize organ regions for focus search by CT in septic patients from 1st to 5th rank. The listed organ regions were as follows: (1) chest or abdomen according to clinical assessment; (2) chest, abdomen, and pelvis together; (3) head, chest, abdomen, and pelvis together; (4) head, neck, chest, abdomen, and pelvis together; and (5) head, neck, chest, abdomen, pelvis, and legs together. Lastly, physicians were asked about their preferred time window for a CT examination in patients diagnosed with or suspected of sepsis. Four options of time windows were offered for selection: (1) < 1 hour, (2) ≥ 1–6 hours, (3) ≥ 6–12 hours, and (4) ≥ 12–24 hours. Participants were additionally asked to indicate their preference for the time window in which potential CT-guided interventions should take place by utilizing the same time window options.

### Administration and data handling

**Fig. 1:**
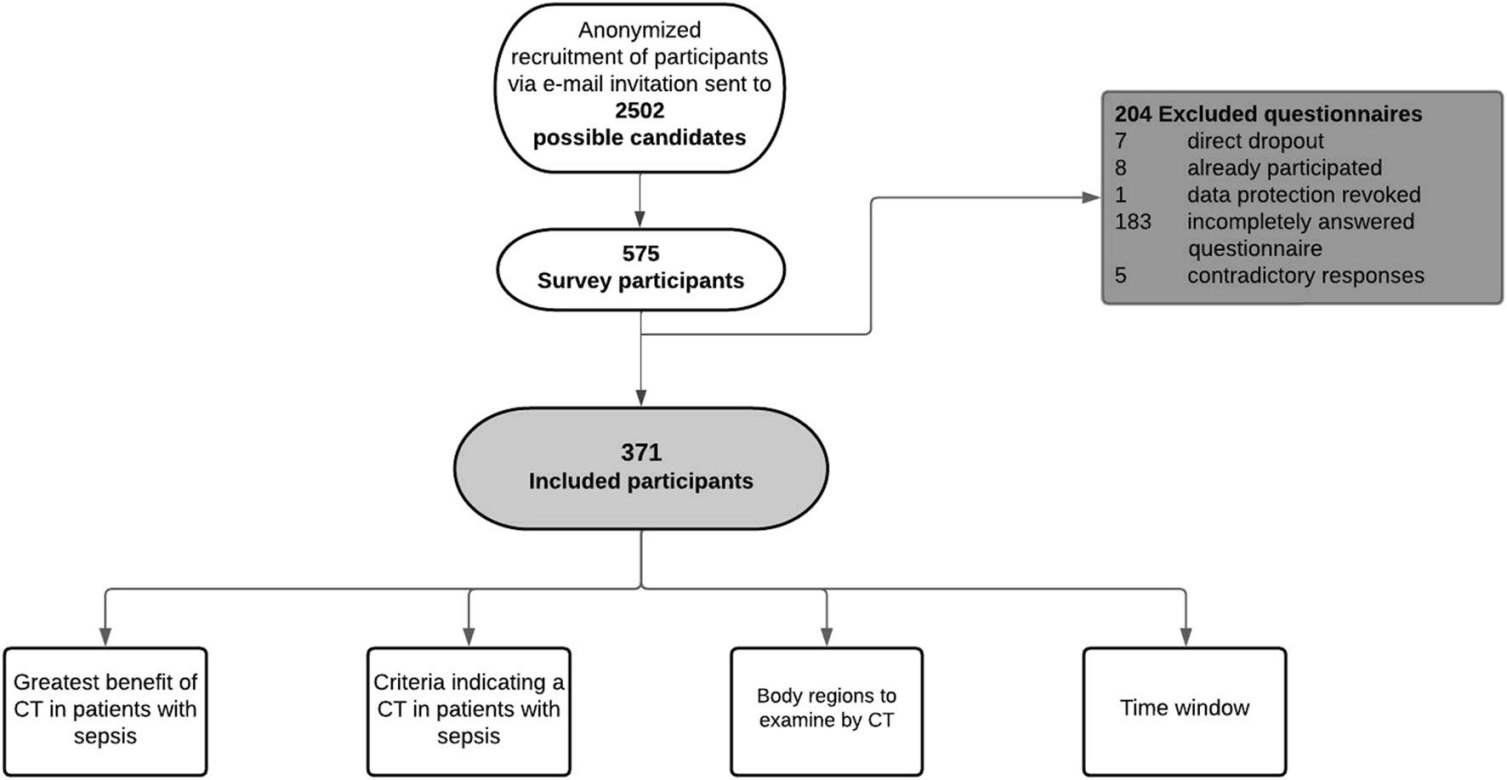
Flow chart of survey participation and questionnaire inclusion and exclusion. Of the 2502 physicians contacted, 575 participated in the survey. After excluding 204 questionnaires, 371 met the inclusion criteria and were included in the analysis

On the 3rd of January 2022, an email containing a link leading to the digital survey was sent through official distribution lists to all physicians at a German university hospital. The link was deactivated on the 31st of January 2022. A total of 2502 physicians were contacted (Fig. 1). Five hundred seventy-five physicians (corresponding to a gross response rate of 23.0%) participated in the study. 64.5% (*n *= 371/575) were included in the analysis (net response rate of 14.8%), as complete responses to all questions were mandatory for inclusion. Only one-time participation was allowed.

### Data analysis

The online platform LimeSurvey (LimeSurvey Cloud, version 5.3.25, 2022; LimeSurvey GmbH) was used for anonymous data collection. Data were extracted to Excel tables (Microsoft® Excel® for Microsoft 365 MSO, version 2112, 2017; Microsoft). Excel was also used for designing graphs. Variables used for statistical analysis were as follows: (a) work experience, (b) workplace, and (c) medical specialty. The three variables can be considered as levels with independent subgroups that are only compared and analyzed within the same level. For example, internal medicine physicians are compared with surgeons (medical specialty) or emergency room physicians with radiology department physicians (workplace). Different numbers of cases were used for the hypothesis analysis, depending on the variable. One participant with two medical specialties was excluded from all analyses with this specific variable to avoid bias. Descriptive statistics such as absolute and relative frequencies and further statistical hypothesis tests such as the chi-square test were performed with SPSS (Statistical Package for the Social Sciences, IBM® SPSS Statistics, version 28.0.1.0, 2021; IBM). The significance level was set to *α *< 0.05. Data are analyzed in an exploratory setting. *p*-values are listed in Table 1.

**Table 1:** Overview of *p*-values obtained by the chi-square test. The responses to each question were analyzed for the influence of the following variables: work experience, workplace, and medical specialty. Significant *p*-values are written in bold. The significance level was set at *α *<  0.05. The supplementary material provides detailed descriptive statistical results for the different variables considered in the clinical and ancillary diagnostic criteria categories

		Work experience	Workplace	Medical specialty
		*p*	*p*	*p*
I see the greatest benefit of CT…	… in confirming the diagnosis of suspected sepsis	0.762	0.055	0.079
	… in the ability to detect a previously unknown focus	0.625	0.536	0.578
	… in the modification of anti-infectious therapy	0.269	0.250	0.631
	… in planning interventions and/or surgeries	0.689	0.076	***0.001***
	… in exclusion diagnosis	0.216	***0.004***	0.207
The following clinical criteria speak for a CT scan:	SOFA score increased by ≥ 2 points	0.229	0.772	0.622
	qSOFA score criteria ≥ 2	0.071	0.156	0.728
	Fever or hypothermia	0.838	***0.005***	***< 0.001***
	SIRS criteria ≥2	0.345	0.082	0.129
	Respiratory rate ≥ 22/min	0.118	0.143	0.295
	Postoperative patient (surgery in the last 7 days)	0.331	0.518	***0.013***
	Increasing catecholamine demand	0.384	0.840	0.648
	Signs of reduced vigilance/altered mental status	0.381	0.837	0.950
	Immunosuppression (due to medication and/or pre-existing illness)	0.121	0.116	***0.020***
	SBP < 100 mmHg or MAP < 65–70 mmHg	0.385	0.075	0.563
	Elderly patient	0.313	0.057	0.063
The following ancillary parameter is of particular relevance for the indication of a CT scan:	Elevated PCT	***0.025***	0.084	0.068
	Elevated CRP	0.644	***0.019***	***< 0.001***
	Leukocytosis or leukopenia	0.475	***0.047***	***0.002***
	Elevated IL-6	0.908	0.248	***0.005***
	Elevated lactate levels	0.756	***< 0.001***	0.620
	Sonographically suspected infection focus	0.263	0.564	0.508
	Abnormal chest x-ray	***0.007***	***0.015***	0.472
	SARS-CoV-2 detected	0.142	0.120	0.121
	Positive blood culture	0.077	***0.028***	0.280
Please prioritize the option of body region to examine by CT in a patient with sepsis (1st rank)		***< 0.001***	***< 0.001***	***0.019***
Time window	What do you consider the best time window for a CT scan after the diagnosis or suspected diagnosis of sepsis?	0.630	0.328	***0.006***
	State your preference regarding the time window after a CT scan in which possible CT-guided interventions (drainage, puncture, etc.) should take place.	0.544	***< 0.001***	***< 0.001***

*CT*, computed tomography; *qSOFA*, Quick Systemic Organ Failure Assessment; *SOFA*, Systemic Organ Failure Assessment; *SIRS*, Systemic Inflammatory Response Syndrome; *SBP*, systolic blood pressure; *MAP*, mean arterial pressure; *PCT*, procalcitonin; *CRP*, C-reactive protein; *IL-6*, interleukin-6; *SARS-CoV-2*, severe acute respiratory syndrome coronavirus type 2

## Results

### Study population

93.3% (*n *= 346/371) of included participants reported dealing with septic patients in their routine clinical practice (Table 2). 35.0% (*n *= 130/371) of the participants had a work experience of > 3 to ≤ 7 years. 51.5% (*n *= 191/371) were assistant physicians. 42.4% (*n *= 157/370) of the participants were internal medicine physicians, while 8.9% (*n *= 33/370) were radiologists. The largest group for the variable “workplace” was the intensive care unit (ICU) with 31.0% (*n *= 115/371), followed by general ward with 22.4% (*n *= 83/371). Table 2 gives a closer look on the demographics and further descriptive data can be found in the supplementary material.

**Table 2:** Demographic data

		*N* = total	Percentage (%)
Work experience *n = 371*	< 3 years	74	19.9
	> 3 – ≤ 7 years	130	35.0
	> 7 – ≤ 11 years	73	19.7
	> 11 – ≤ 20 years	64	17.3
	> 20 years	30	8.1
Position *n= 371*	Assistant physician	191	51.5
	Board-certified physician	99	26.7
	Senior or chief physician	81	21.8
Medical specialty *n = 370*	Internal medicine	157	42.4
	Surgery	44	11.9
	Anesthesiology	70	18.9
	Radiology	33	8.9
	Other specialty	66	17.8
Workplace (>50% of working time) *n = 371*	Intensive care unit	115	31.0
	General ward	83	22.4
	Emergency department	49	13.2
	Operating room (OR)	42	11.3
	Radiology	32	8.6
	Outpatient clinic	32	8.6
	Others	18	4.6
Sepsis-related fellowship *n = 225*	Hospital hygiene	1	0.4
	Infectiology	9	4.0
	Clinical acute and emergency medicine	13	5.8
	Emergency medicine	68	30.2
	Intensive care medicine	75	33.4
	Qualification: specialist knowledge in radiation protection	59	26.2
Do you deal with septic patients in your daily clinical practice? *n = 371*	Yes	346	93.3
	No	25	6.7

### Expected benefits of CT in septic patients

**Fig. 2:**
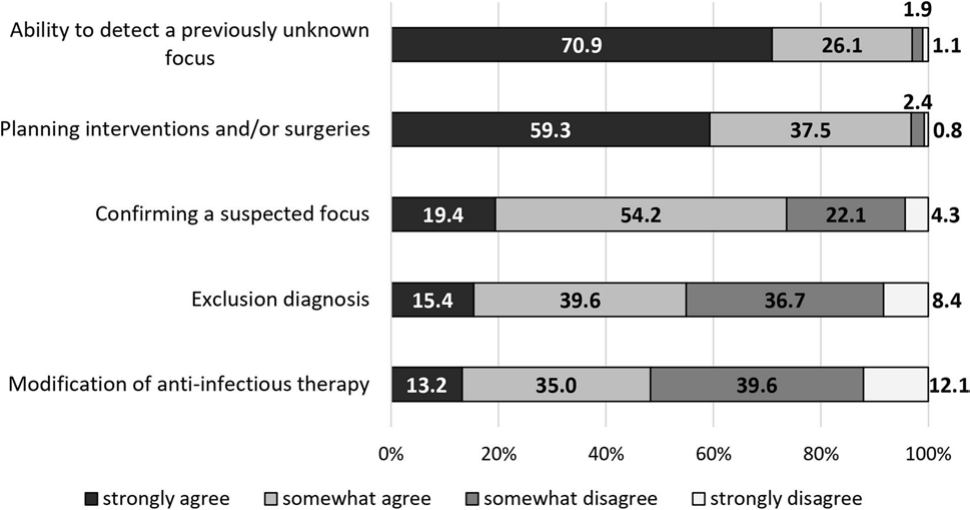
Arguments in favor of a CT scan in sepsis — why should a patient be examined by CT? Overview of results for whether physicians believe the listed options to represent a major benefit of CT examinations in septic patients. With 97.0% (*n *= 360/371, “strongly agree” and “somewhat agree” counted together), the majority of participants agreed that the ability of CT to detect an unknown focus is a great benefit, followed by 96.8% (*n *= 359/371) of respondents seeing a great advantage of CT in planning interventions and/or surgeries. CT to confirm a suspected focus was considered a major advantage by 73.6% (*n *= 273/371). 55.0% (*n *= 204/371) of participants considered CT to be of great benefit as a diagnostic tool for exclusion. The modification of anti-infective therapy after a CT scan was the option most disagreed with: 51.7% (*n *= 192/371) of physicians ticked the options “strongly disagree” or “somewhat disagree.” CT, computed tomography

70.9% (*n *= 263/371) of participants fully agreed that the ability of CT to detect an unknown focus is a great benefit of CT scans (Fig. 2). The modification of anti-infective therapy after CT examination was the option most likely to not be considered a major benefit of CT (51.7%, *n *= 192/371). 59.3% of physicians (*n *= 220/371) fully agreed on seeing a great advantage of CT in planning interventions and/or surgeries. Here, a difference was found between surgeons versus (a) internal medicine physicians and (b) anesthesiologists (Table 1). While 81.8% (*n *= 36/44) of surgeons saw one of the greatest benefits of CT in patients with sepsis in the ability to plan interventions and/or surgeries based on a scan, only 40.0% (*n *= 28/70) of anesthesiologists and 56.1% (*n *= 88/157) of internal medicine physicians fully agreed regarding this item. CT for ruling-out a suspected infectious focus was considered likely a benefit by 39.6% (*n *= 147/371), while 36.7% (*n *= 136/371) did not quite agree (Fig. 2). Here, a significant difference was detectable according to participants’ workplace (Table 1). “Strongly disagree” was most frequently reported by physicians from emergency (24.0%, *n *= 12/50) and radiology department (18.8%, *n *= 6/32), while OR physicians not once ticked this answer option (0.0%, *n *= 0/42). When looking at the medical specialty, radiologists (60.6%, *n *= 20/33) together with surgeons (65.9%, *n *= 29/44) were the groups most likely to agree on the benefit of exclusion diagnosis through CT.

### Relevance of clinical and ancillary diagnostic criteria — relevant for a CT indication

**Fig. 3:**
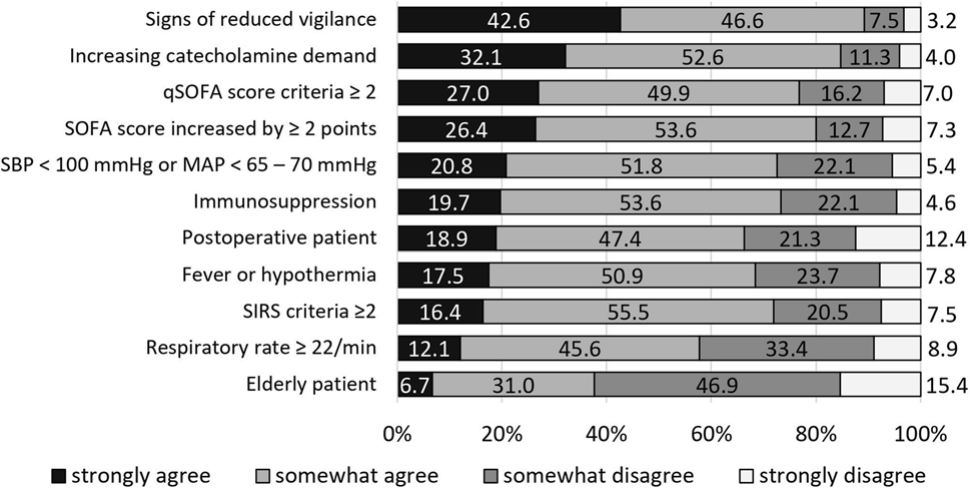
Descriptive results for physicians’ responses regarding whether they considered the listed clinical criteria to support a CT request in patients with sepsis. Except for the “elderly patient” criterion, each clinical criterion listed was considered by more than 50% of the participants as a reasonable argument in favor of a CT scan in patients with sepsis. The criterion that most physicians selected to support a CT request was “signs of decreased vigilance” (89.2%, *n *= 331/371), followed by “increased catecholamine demand” (84.7%, *n *= 314/371). A critical qSOFA and a critical SOFA score were similarly classified. Whereas a qSOFA score with ≥ 2 applicable criteria was considered relevant by 76.9% (*n *= 285/371) of participants, a SOFA score increased by ≥ 2 points was considered relevant by 80.0% (*n *= 297/371). At 55.5% (*n *= 206/371), the SIRS criteria were most frequently classified as only somewhat relevant. Significant levels (*p*-values) for response patterns in relation to work experience, workplace, and medical specialty are compiled in Table 1. Detailed frequency data for the analyzed variables can be found in the supplementary material. CT, computed tomography; qSOFA, Quick Systemic Organ Failure Assessment; SOFA, Systemic Organ Failure Assessment; SBP, systolic blood pressure; MAP, mean arterial pressure; SIRS, Systemic Inflammatory Response Syndrome

Most participants, i.e., a median of 72.6% (Q1 = 66.3, Q3 = 80.0), considered the listed clinical criteria to be an argument for a CT scan in patients with sepsis (Fig. 3). The criterion “elderly patient,” which 62.3% (*n *= 231/371) disagreed with, emerged as an outlier. A sign of decreased vigilance was the most strongly agreed upon criterion for requesting CT diagnostics (42.6%, *n *= 158/371). More than 50% of the participants considered each of the queried screening tools — Systemic Organ Failure Assessment (SOFA) and Quick Systemic Organ Failure Assessment (qSOFA) scores, and Systemic Inflammatory Response Syndrome (SIRS) criteria — as a relevant CT indication (Fig. 3).

**Fig. 4:**
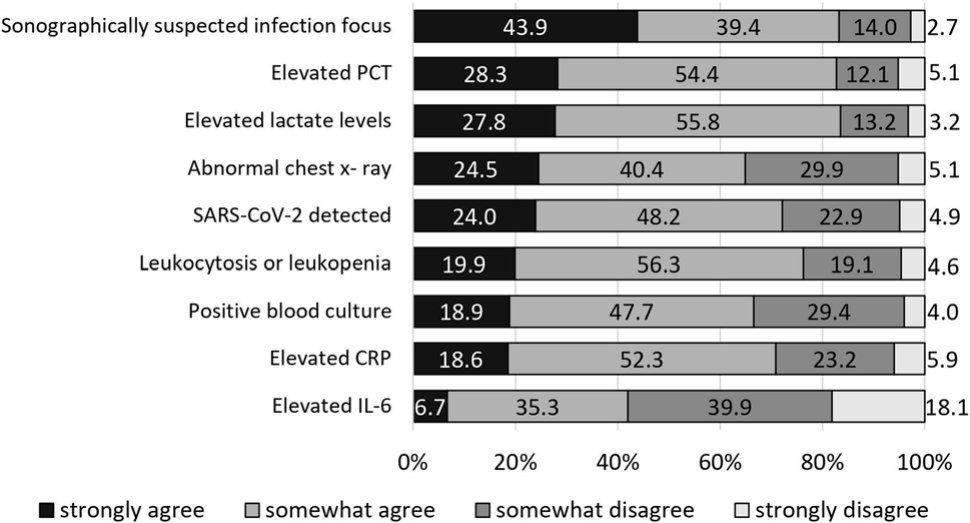
Descriptive results for physicians’ responses regarding whether they considered the listed ancillary criteria to support a CT request in patients with sepsis. Although a sonographically suspected focus of infection was considered an indication for a CT scan by more participants (83.3%, *n *= 309/371) than an abnormal chest x-ray (64.9%, *n *= 241/371), a general desire for confirmation of previous imaging findings by CT is apparent. More than half of physicians would also request CT in patients with evidence of SARS-CoV-2 (72.2%, *n *= 268/371) and a positive blood culture (66.6%, *n *= 247/371). Of the laboratory parameters listed, elevated PCT (82.7%, *n*=307/371) and elevated lactate (83.6%, *n*=310/371) were considered the strongest arguments for requesting a CT. The leukocyte count (76.2%, *n *= 282/371) and elevated CRP (70.9%, *n *= 263/371) were also selected by physicians as reasons for a CT request. Conversely, the criterion “elevated IL-6” was not considered a criterion supporting a CT indication by 58% (*n *= 215/371). No difference was found in the response pattern for “sonographically suspected focus of infection” and “SARS-CoV-2 detected.” Regardless of the variables examined, all physicians marked these parameters as supportive of a CT scan. Significant levels (*p*-values) for response patterns in relation to work experience, workplace, and medical specialty are stated in Table 1. Detailed frequency data for the analyzed variables can be found in the supplementary material. CT, computed tomography; PCT, procalcitonin; SARS-CoV-2, severe acute respiratory syndrome coronavirus type 2; CRP, C-reactive protein; IL-6, interleukin-6

The majority of participants considered each of the listed ancillary criteria as a relevant parameter that supports performing a CT scan in a septic patient (median 72.2%; Q1 = 65.8, Q3 = 83.0) (Fig. 4). A sonographically suspected focus of infection was considered the strongest ancillary parameter for a CT indication (83.3%, *n *= 309/371). An abnormal chest x-ray was also considered relevant for a CT request by the majority (64.9%, *n *= 241/371). Here, significant differences were detectable in the subgroups of work experience and workplace (Table 1). Contrary, each medical specialty subgroup rather agreed that an abnormal chest x-ray can be seen as indication for a CT in septic patients (Supplementary material). The laboratory parameters listed — particularly elevated PCT and lactate levels — were indicated as relevant for CT indication (Fig. 4). “Elevated IL-6” was an outlying parameter, which 58.0% (*n *= 215/371) disagreed with. Detailed statistics regarding the clinical and ancillary diagnostic criteria can be found in the supplementary material.

### Body regions — what to cover with one scan

Overall, 42.6% (*n *= 158/371) of the participants prioritized the examination of the chest or abdomen according to clinical presentation. However, 34.8% (*n *= 129/371) prioritized examining the chest, abdomen, and pelvis altogether (Table 3). Surgeons preferred the combined examination of the chest, abdomen, and pelvis in septic patients, whereas internal medicine physicians preferred a CT scan of either chest or abdomen (Table 4). Further analysis of the responses for the first rank identified differences for all three variables (Tables 1 and 4). With regard to the working place, only physicians working at the ICU favored a simultaneous examination of the chest, abdomen, and pelvis.

**Table 3:** Prioritization of organ regions to be examined for focus search by CT in sepsis. The more body regions were examined at once, the less often the participants prioritized the option. Most physicians (42.6%, *n *= 158/371) would order a CT scan of the chest or abdomen according to clinical assessment. The second most frequently selected option as the first rank was the simultaneous examination of the chest, abdomen, and pelvis (34.8%, *n *= 129/371). A whole-body CT scan (head/neck/chest/abdomen/pelvis/legs) was chosen as the first rank by only 2.2% (*n *= 8/371) and as the fifth rank by 63.9% (*n *= 237/371)

	Chest or abdomen according to clinical assessment		Chest and abdomen and pelvis		Head and chest and abdomen and pelvis		Head and neck and chest and abdomen and pelvis		Head and neck and chest and abdomen and pelvis and legs	
	*n*	%	*n*	%	*n*	%	*n*	%	*n*	%
1st rank	158	42.6	129	34.8	41	11.1	35	9.4	8	2.2
2nd rank	91	24.5	150	40.4	77	20.8	34	9.2	19	5.1
3rd rank	41	11.1	44	11.9	185	49.9	71	19.1	30	8.1
4th rank	39	10.5	30	8.1	48	12.9	177	47.7	77	20.8
5th rank	42	11.3	18	4.9	20	5.4	54	14.6	237	63.9

*CT*, computed tomography

**Table 4:** Detailed frequency data for the analyzed variables regarding the first choice (1st rank) of body regions to examine with CT. Results are given as percentages with absolute numbers in parentheses. Work experience had a significant effect on the body regions preferred for a CT examination (*p *< 0.001, chi-square test). While physicians with < 3 years of experience never prioritized the option of a whole-body CT scan from head to legs; physicians with more experience tended to choose that option more often — with 17.0% (*n *= 5/30) in the group with >20 and 3.0% (*n *= 2/64) in the one with >11 to ≤ 20 years of experience. Significant differences were also found between workplaces (*p *< 0.001, chi-square test). ICU physicians were the only group that prioritized (37.7%, *n *= 41/115) the simultaneous examination of chest, abdomen, and pelvis altogether. In contrast, physicians working in general wards, the emergency department, and operation room mainly chose a CT examination of the chest or abdomen according to clinical assessment as the first rank. Regarding the influence of medical specialties on the choice of body regions to be examined by CT, surgeons most frequently (52.3%, *n *= 23/44) opted for the simultaneous examination of the chest, abdomen, and pelvis (*p *= 0.019, chi-square test). Radiologists preferred the simultaneous examination of either the chest, abdomen, and pelvis or of the head, chest, abdomen, and pelvis (24.2% or *n *= 8/33, each)

		Chest or abdomen according to clinical assessment	Chest and abdomen and pelvis	Head and chest and abdomen and pelvis	Head and neck and chest and abdomen and pelvis	Head and neck and chest and abdomen and pelvis and legs
Work experience in years (*n*=371)	< 3 (*n *= 74)	54.1 (40)	33.8 (25)	9.5 (7)	2.7 (2)	0.0 (0)
	> 3 – ≤ 7 (*n *= 130)	42.3 (55)	38.5 (50)	9.2 (12)	10.0 (13)	0.0 (0)
	> 7 – ≤ 11 (*n *= 73)	39.7 (29)	32.9 (24)	16.4 (12)	9.6 (7)	1.4 (1)
	> 11 – ≤ 20 (*n *= 64)	34.4 (22)	34.4 (22)	12.5 (8)	15.6 (10)	3.1 (2)
	> 20 (*n *= 30)	40.0 (12)	26.7 (8)	6.7 (2)	10.0 (3)	16.7 (5)
Workplace (*n*=371)	ICU (*n*=115)	28.7 (33)	35.7 (41)	20.0 (23)	12.2 (14)	3.5 (4)
	General ward (*n *= 82)	54.9 (45)	36.6 (30)	6.1 (5)	2.4 (2)	0.0 (0)
	Emergency department (*n *= 50)	54.0 (27)	34.0 (17)	6.0 (3)	6.0 (3)	0.0 (0)
	OR (*n *= 42)	52.4 (22)	38.1 (16)	2.4 (1)	2.4 (1)	4.8 (2)
	Radiology (*n *= 32)	40.6 (13)	25.0 (8)	21.9 (7)	12.5 (4)	0.0 (0)
	Outpatient clinic (*n *= 32)	40.6 (13)	31.3 (10)	0.0 (0)	21.9 (7)	6.3 (2)
	Other (*n *= 18)	27.8 (5)	38.9 (7)	11.1 (2)	22.2 (4)	0.0 (0)
Medical specialty (*n*=370)	Internal medicine (*n *= 157)	47.1 (74)	33.1 (52)	12.1 (19)	5.1 (8)	2.5 (4)
	Surgery (*n *= 44)	38.6 (17)	52.3 (23)	2.3 (1)	2.3 (1)	4.5 (2)
	Radiology (*n *= 33)	39.4 (13)	24.2 (8)	24.2 (8)	12.1 (4)	0.0 (0)
	Anesthesiology (*n *= 70)	38.6 (27)	34.3 (24)	10.0 (7)	15.7 (11)	1.4 (1)
	Other (*n *= 66)	39.4 (26)	33.3 (22)	9.1 (6)	16.7 (11)	1.5 (1)

*CT*, computed tomography; *ICU*, intensive care unit; *OR*, operating room

### Time window for CT examination and CT-guided intervention in sepsis

**Fig. 5:**
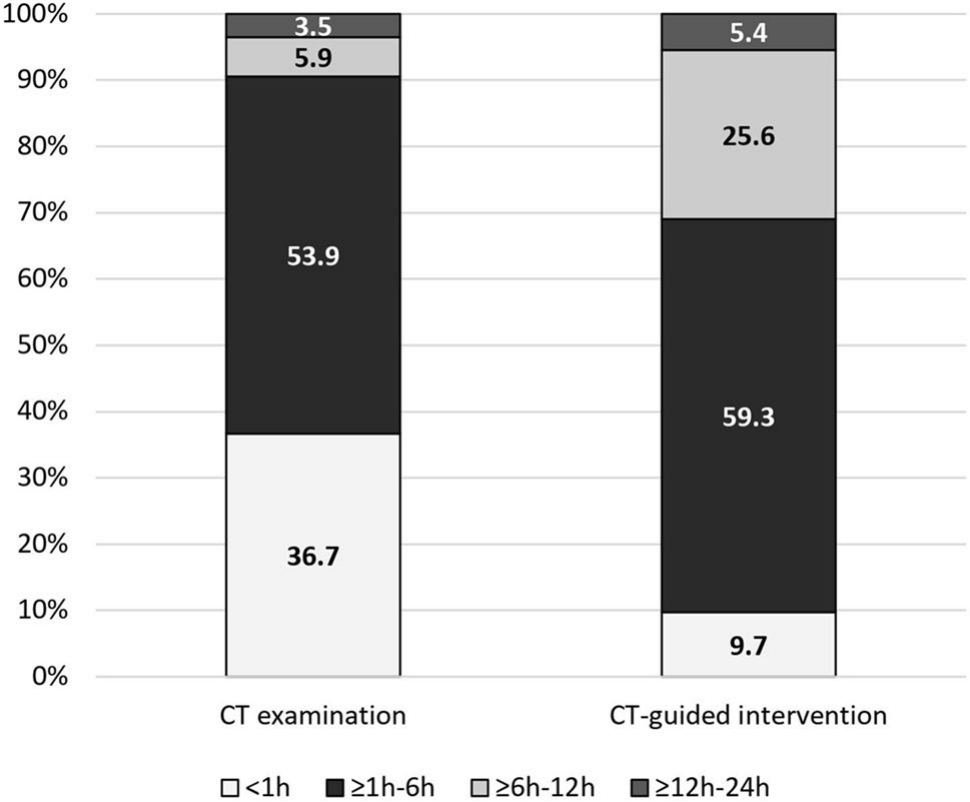
Time window for CT examinations and CT-guided interventions in patients with sepsis. The ≥ 1–6 h time window was most frequently selected by participants for both CT examinations for suspected sepsis (53.9%, *n *= 200/371) and CT-guided interventions (59.3%, *n *= 220/371). While the second most frequently chosen time window for a CT scan was <1h (36.7%, *n *= 136/371), it was the ≥ 6–12h time window for CT-guided interventions with 25.6% (*n *= 95/371). Conversely, only 9.7% (*n *= 36/371) of physicians indicated a preference for CT-guided interventions within 1 hour, and 5.9% (*n *= 22/371) for CT examinations within 6 to 12 hours. The time window ≥ 12–24 h for both a CT scan (3.5%, *n *= 13/371) and a CT-guided intervention (5.4%, *n *= 20/371) was the least frequently ticked option. CT, computed tomography; h, hour/hours

When asked about the preferred time window for a CT scan after diagnosis or suspicion of sepsis, overall, 53.9% (*n *= 200/371) of the participants chose ≥ 1–6 hours (h). With 36.7% (*n *= 136/371), the second most frequently chosen time window was < 1h (Fig. 5). With regard to the workplace, physicians from the radiology department were the least likely to choose the < 1 h time window (15.6%, *n *= 5/32) for a CT examination, while it was the time window preferred by emergency department physicians (50%, *n *= 25/50). There were significant differences in the responses between medical specialties, with only 18.2% (*n *= 6/33) of radiologists but 59.1% (*n *= 26/44) of surgeons choosing the shortest time window of < 1 h (Table 1).

**Fig. 6:**
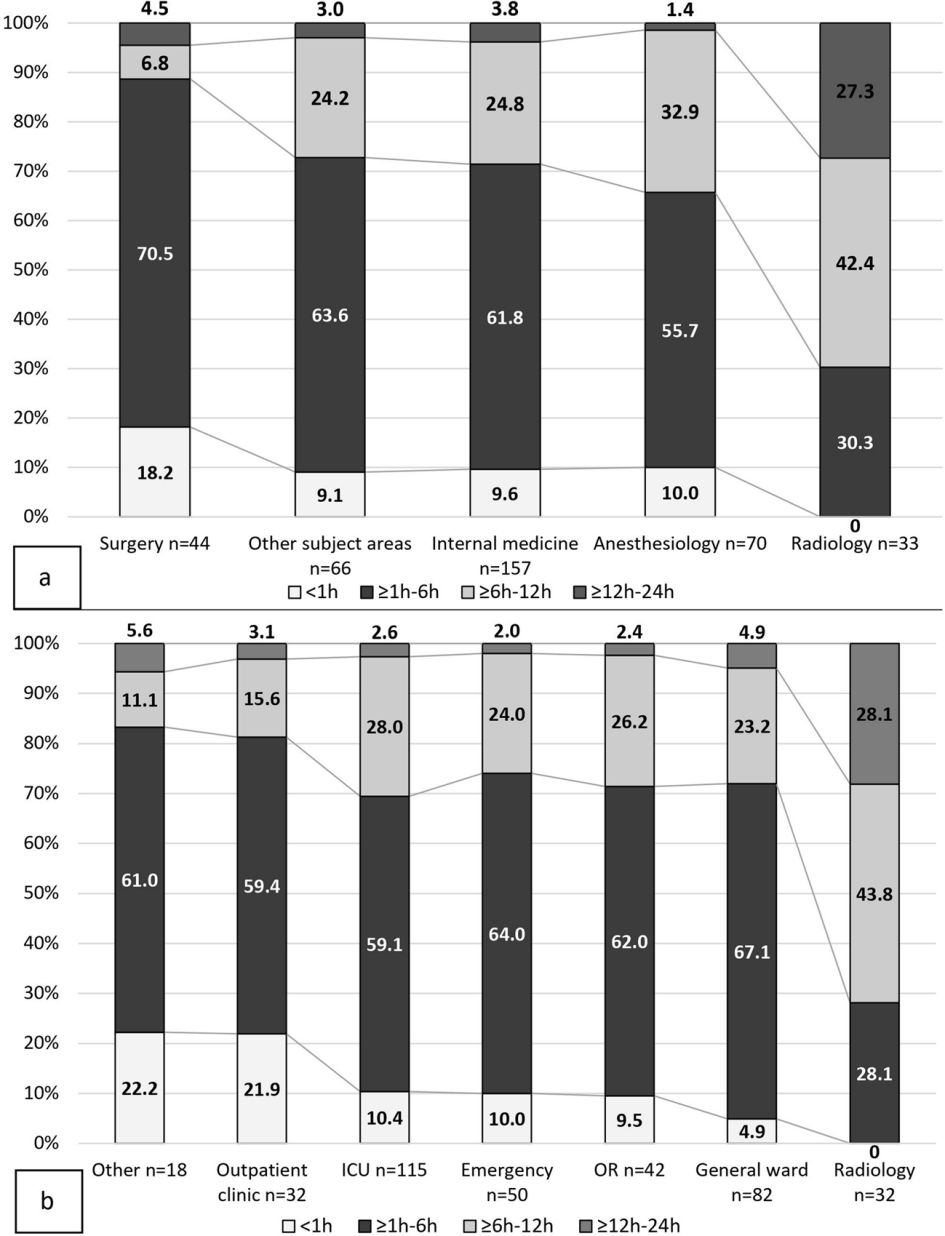
**a** Time window for CT interventions and effect of medical specialty. Significant differences (*p *< 0.001, chi-square test) were found between radiologists and each of the other four medical specialties. While the other medical specialties preferred the time window of ≥ 1–6 h, radiologists mainly chose the time window of ≥ 6–12 h with 42.4% (*n *= 14/33). While the option of ≥ 12–24 h was chosen by 27.3% (*n *= 9/33) of radiologists, surgeons were the group most likely to choose this option among the other medical specialties, but at only 4.5% (*n *= 2/44). The time window of < 1h was not selected once by radiologists. Conversely, the option to have a CT intervention within 1 hour was chosen by 18.2% (*n *= 8/44) of surgeons. **b** Time window for CT-guided interventions and effect of the workplace. Significant differences (*p *< 0.001, chi-square test) were found between the radiology department and each of the other workplaces. The majority of physicians preferred a time window of ≥ 1–6 h for a CT-guided intervention. Radiologists, on the other hand, preferred the ≥ 6–12 h time window (43.8%, *n *= 14/32). While the option of ≥ 12–24 h was chosen by 28.1% (*n *= 9/32) of radiologists, only one physician each from the emergency department (2.0%), OR (2.4%), outpatient clinic (3.1%), and other (5.6%) chose this option. Only radiologists never selected the time window of < 1 hour even once. CT, computed tomography; ICU, intensive care unit; OR, operating room; h, hour/hours

When asked about the preferred time window for a CT-guided intervention after focus detection by CT, overall, 59.3% (*n *= 220/371) of all physicians chose the time window ≥ 1–6 h, with 25.6% (*n *= 95/371) opting for the second most widely chosen time window of ≥ 6–12 h (Fig. 5). Statistically significant differences were found between the medical specialties (Table 1; Fig. 6a) and the various workplaces (Table 1; Fig. 6b).

## Discussion

### Summary

In this study, physicians described the greatest benefit of CT scans in patients with sepsis as the ability to detect a previously unknown infectious source and the ability to plan interventions and/or surgery based on CT findings. The clinical criteria that most physicians considered strongly relevant for requesting a CT examination were signs of decreased vigilance and an increased catecholamine demand. Of the laboratory parameters, elevated procalcitonin and lactate levels were considered the most decisive for CT indication. Based on clinical findings, most physicians would order a CT scan of the chest or abdomen. A CT scan including the chest, abdomen, and pelvis was the first choice in cases without clinical evidence of a focus. For both a CT examination in patients with suspected sepsis and a CT-guided intervention, a time window ≥ 1–6 h was preferred by responders. In both questions, radiologists selected the time window of < 1h least frequently. Conversely, emergency department physicians preferred a CT examination in < 1h.

### Literature

To the best of our knowledge, this is the first study to analyze physicians’ perspectives on the use of CT in patients with sepsis. Between 2003 and 2015, the incidence rate in high-income countries was an estimated 707 sepsis cases per 100,000 person-years [16]. This high incidence of sepsis is indirectly reflected in our data. The group of participants who do not deal with septic patients in daily clinical practice was remarkably small at 6.7%. With a reported range of 95 to 97%, the exclusion diagnosis by CT was rated as helpful in confirming or ruling out an alternative diagnosis in the emergency department setting [15]. In our study, CT for exclusion diagnosis was also considered to be beneficial. Overall, the heterogeneous response pattern between “somewhat agree” and “somewhat disagree” among our participants suggests a difference of opinion. Physicians in the emergency department disagreed the most (24%, *n *= 12/50) that an exclusion diagnosis is a major benefit of CT scans. The fact that fewer physicians in our survey considered exclusion by CT useful may be explained by the different patient groups. In our survey, we are not dealing only with emergency department patients. Additionally, we solely focused on septic patients, while Pandharipande et al focused on patients with abdominal or chest pain or headache [15]. Furthermore, 18.8% (*n*=6/32) of radiologists in our survey strongly disagreed that one of the greatest benefits of CT in patients with sepsis is exclusion diagnosis. The SSC guidelines do not contrast the benefits of CT imaging in septic patients with the side effects, such as radiation exposure [2].

While radiation protection must always be considered when CT is used [17, 18], a clinically indicated CT examination should not be postponed or declined out of “fear of radiation” [9]. Fittingly, a large proportion of our study participants saw a CT indication for closer examination in patients with a sonographically suspected septic focus or an abnormal x-ray finding. It is noteworthy that none of the variables analyzed seemed to have a significant impact on the physicians’ responses: the sonographically suspected focus was considered as an indication for a CT scan by the vast majority. In contrast, significant differences were detected in the parameter “abnormal chest X-ray findings” between the different work experiences and workplaces. However, when considering the variable medical specialty, a fairly homogeneous response pattern was found with only small, insignificant differences in the response pattern. In general, each medical specialty group agreed that an abnormal chest x-ray can be seen as indication for a CT in septic patients. This resembles a possible desire for more objective and accurate evidence of septic foci through CT. Overall, confirmation of a suspected focus or identifying a previously unknown focus was considered an advantage of CT in patients with sepsis. The identification or exclusion of an infectious focus in septic patients, especially when the focus is unknown, is not only considered a major advantage of CT by the participants but is also recommended in the guidelines [2]. Another imaging modality used for anatomic search of unknown foci includes the combined examination through positron emission tomography and CT (PET-CT) in, e.g., patients with fever of unknown origin (FUO) [19]. Even though this is beyond the scope of the study, a study on the role and possibilities of PET-CT examination in patients with sepsis would be interesting.

The ability to modify anti-infective therapy was considered a benefit of CT by the lowest percentage of responders (13.2%, *n *= 49/371). Administration of anti-infective medications as soon as possible is recommended for infectious source control [2]. Delayed initiation of anti-infective therapy leads to higher in-hospital mortality [20, 21]. However, once the cause of sepsis has been identified, the initially broadly applied anti-infectives should be de-escalated, if possible, to targeted therapy [22]. A CT scan can help in identifying the focus and source of infection, allowing for a switch in the anti-infective regimen, as shown by Schleder et al [11].

An intervention no later than 6–12 hours after diagnosis is beneficial and thus should not be delayed without a good reason [2, 3, 8]. The optimal type of intervention remains to be defined. The time window of ≥1–6 hours was preferred in our study, and interventions later than 12 hours did not seem desirable.

The body regions for which participants would order a CT scan are consistent with published data. The chest, abdomen, and pelvis are the body regions most commonly examined by CT and the most common location of infectious sources [11–13, 20, 22]. Surgeons tended towards requesting a joint examination of the thorax, abdomen, and pelvis. The simultaneous examination of the three regions could be related to the fact that many surgeons are aware of the relevance of postoperative complications but also consider the typical areas for sepsis foci. Many surgeons considered CT an advantage for exclusion diagnosis. Scanning the above-mentioned regions where sepsis foci are most frequently located might show a desire for simultaneous precise and broad focus search.

Although tools such as the SIRS criteria and the qSOFA score should no longer be used as the sole screening tools for sepsis, screening of critically ill patients for sepsis is still recommended as they allow the fastest possible identification of septic patients [2, 23]. The screening tools used in clinical practice, such as the SOFA and qSOFA scores, and the SIRS criteria with values suggestive of sepsis or infection, have also been interpreted as an indication for a CT scan by most physicians in our survey.

Patients with sepsis or suspected sepsis are at risk of septic shock. One criterion for a septic shock is an elevated lactate level [24]. Lactate has been described as a biomarker of critical organ perfusion [10, 22]. In our survey, the laboratory value “elevated lactate levels” was most frequently chosen (83.6%, *n *= 310/371) as an argument for a CT in patients with sepsis or suspected sepsis. Other studies have not described elevated lactate levels as indicative of CT. Consistent with published findings, participating physicians in our survey consider elevated lactate levels to be of concern, leading to the wish to perform a CT to detect an infectious focus as soon as possible. The SSC 2021 recommends measuring lactate levels in patients with suspected sepsis but also emphasizes that this parameter alone is insufficient to confirm or rule out the diagnosis of sepsis [2]. The pro-inflammatory biomarkers CRP and PCT are recommended and widely used as diagnostic tools and for monitoring infection and inflammation in patients with sepsis, especially when used in conjunction with clinical assessment and determination of other biomarkers [2, 10, 25–27]. In this regard, PCT has been described as a more sensitive and accurate biomarker than CRP [10, 27]. Fittingly, more physicians considered elevated PCT rather than elevated CRP levels to be more important in deciding the need for a CT in our study. Consistent with studies recommending simultaneous consideration of multiple biomarkers rather than just one [26, 27], respondents in our study predominantly agreed with all of the listed laboratory parameters supporting a CT request.

Of note, most of the studies mentioned refer to the diagnosis of sepsis and not the use of CT in patients with sepsis. Therefore, comparisons are limited.

Radiologists do not request CTs in clinical practice. However, they are important partners in discussing the indication with treating physicians and thus can influence details of CT requests (in terms of body region, use of contrast medium, etc.). It would be interesting to see whether radiologists place different emphases on the indication for CT examinations in septic patients than the clinicians who typically request CTs. To investigate more precisely the differences between radiologists and non-radiologists, a larger number of radiologists should be surveyed. A comprehensive analysis of the radiologist’s perspective on CT in sepsis is beyond the scope of this manuscript and will be reported elsewhere.

### Limitations

The response rate in this survey can be considered a limitation, although the number of participants exceeded the expected response rate. The results of our survey present the perspective of physicians of a single large university hospital and do not necessarily reflect the situation in other hospitals and other countries. However, we have considered and mapped the heterogeneity of all physician groups involved with septic patients. Moreover, the high number of physicians stating that they were involved in managing patients with sepsis in their daily clinical practice suggests that our data reflect opinions based on practical experience. The large number of assistant physicians participating can be seen as a limitation, as the distribution of the positions (1) assistant, (2) board-certified, and (3) senior/chief physicians is not one-third each. However, this reflects the local environment of the hierarchical staffing structure of university hospitals. In the analysis, we decided to use the work experience in years as a variable instead of physician’s position. Finally, the fact that closed-ended questions (Likert scales) with limited response options were used rather than open-ended questions could be considered a limitation of the questionnaire. However, to reduce the influence on participants’ responses, we avoided leading-type questions. To curb the tendency to choose the middle of the response spectrum, we designed a 4-point Likert scale.

## Conclusion

CT is considered a pivotal tool in critically ill patients with (suspected) sepsis and is widely used in clinical practice. Despite a lack of guidelines, there is considerable overlap in this survey’s participants’ responses on many aspects concerning the use of CT in patients with sepsis. However, we found statistically significant differences between workplaces and medical specialties regarding crucial aspects. These data may improve interdisciplinary communication about imaging decisions in sepsis. The optimal timing of CT in sepsis should be analyzed further in a prospective setting. Evidence-based imaging guidelines should be established to achieve the best patient outcome and avoid losing time in treating sepsis.

### Supplementary information

ESM 1 (PDF 413 kb)

## Abbreviations

CRP: C-reactive protein
CT: Computed tomography
CXR: Chest radiography
h: Hour/hours
ICU: Intensive care unit
IL-6: Interleukin-6
MAP: Mean arterial pressure
OR: Operating room
PCT: Procalcitonin
PET-CT: Positron emission tomography and computed tomography
Q1 and Q3: First quartile and third quartile
qSOFA: Quick Systemic Organ Failure Assessment
SARS-CoV-2: Severe acute respiratory syndrome coronavirus type 2
SBP: Systolic blood pressure
SIRS: Systemic Inflammatory Response Syndrome
SOFA: Systemic Organ Failure Assessment
SSC: Surviving Sepsis Campaign
USG: Ultrasonography

## Declarations

### Guarantor

The scientific guarantor of this publication is Julian Pohlan.

### Statistics and biometry

One of the authors has significant statistical expertise.

### Methodology

- prospective
- experimental
- performed at one institution

## References

[1] Singer M, Deutschman CS, Seymour CW (2016). The Third International Consensus Definitions for Sepsis and Septic Shock (Sepsis-3). JAMA..

[2] Evans L, Rhodes A, Alhazzani W, Antonelli M (2021). Surviving Sepsis Campaign: International Guidelines for Management of Sepsis and Septic Shock 2021. Crit Care Med..

[3] Arbeitsgemeinschaft der Wissenschaftlichen Medizinischen Fachgesellschaften (2018) S3-Leitlinie Sepsis – Prävention, Diagnose, Therapie und Nachsorge. Arbeitsgemeinschaft der Wissenschaftlichen Medizinischen Fachgesellschaften, Frankfurt am Main. Available via https://www.awmf.org/uploads/tx_szleitlinien/079-001l_S3_Sepsis-Praevention-Diagnose-Therapie-Nachsorge_2020-03_01.pdf. Accessed 18 Sept 2021

[4] Napolitano LM (2018). Sepsis 2018: Definitions and Guideline Changes. Surg Infect (Larchmt).

[5] Oliver ZP, Perkins J (2017). Source identification and source control. Emerg Med Clin North Am..

[6] Rhodes A, Evans LE, Alhazzani W (2017). Surviving Sepsis Campaign: International Guidelines for Management of Sepsis and Septic Shock: 2016. Intensive Care Med..

[7] Dellinger RP, Levy MM, Rhodes A (2013). Surviving Sepsis Campaign Guidelines Committee including the Pediatric Subgroup. Surviving sepsis campaign: international guidelines for management of severe sepsis and septic shock: 2012. Crit Care Med..

[8] De Waele JJ, Sakr Y (2019). How I search for a sepsis source. Crit Care..

[9] German Commission on Radiological Protection (2019) Recommendations for medical imaging procedures. Federal Ministry for the Environment, Nature Conservation, Nuclear Safety, and Consumer Protection Bonn. Available via https://www.ssk.de/SharedDocs/Beratungsergebnisse_PDF/2019/2019-06-27Orientie_e.pdf?__blob=publicationFile. Accessed 29 May 2022

[10] Rello J, Valenzuela-Sánchez F, Ruiz-Rodriguez M, Moyano S (2017). Sepsis: A Review of Advances in Management. Adv Ther..

[11] Schleder S, Luerken L, Dendl LM (2017). Impact of multidetector computed tomography on the diagnosis and treatment of patients with systemic inflammatory response syndrome or sepsis. Eur Radiol..

[12] Pohlan J, Witham D, Opper Hernando MI et al (2022) Relevance of CT for the detection of septic foci: diagnostic performance in a retrospective cohort of medical intensive care patients. Clin Radiol 77(3):203–209. 10.1016/j.crad.2021.10.020 10.1016/j.crad.2021.10.020 34872706

[13] Pohlan J, Hernando MIO, Hogrebe A et al (2020) The role of body computed tomography in hospitalized patients with obscure infection: Retrospective consecutive cohort study. Eur J Radiol 132:109325. 10.1016/j.ejrad.2020.109325 10.1016/j.ejrad.2020.109325 33027726

[14] Pohlan J, Witham D, Muench G et al (2021) Computed tomography for detection of septic foci: Retrospective analysis of patients presenting to the emergency department. Clin Imaging 69:223–227. 10.1016/j.clinimag.2020.09.004 10.1016/j.clinimag.2020.09.004 32971451

[15] Pandharipande PV, Reisner AT, Binder WD (2016). CT in the Emergency Department: A Real-Time Study of Changes in Physician Decision Making. Radiology..

[16] Fleischmann C, Scherag A, Adhikari NK (2016). Assessment of Global Incidence and Mortality of Hospital-treated Sepsis. Current Estimates and Limitations. Am J Respir Crit Care Med..

[17] Padole A, Ali Khawaja RD, Kalra MK, Singh S (2015). CT radiation dose and iterative reconstruction techniques. AJR Am J Roentgenol..

[18] Ditchfield M (2016). CT and radiation dose: Where are we now?. J Med Imaging Radiat Oncol..

[19] Kouijzer IJE, Mulders-Manders CM, Bleeker-Rovers CP, Oyen WJG (2018). Fever of Unknown Origin: the Value of FDG-PET/CT. Semin Nucl Med..

[20] Ferrer R, Martin-Loeches I, Phillips G (2014). Empiric antibiotic treatment reduces mortality in severe sepsis and septic shock from the first hour: results from a guideline-based performance improvement program. Crit Care Med..

[21] Liu VX, Fielding-Singh V, Greene JD, Baker JM, Iwashyna TJ, Bhattacharya J, Escobar GJ (2017). The Timing of Early Antibiotics and Hospital Mortality in Sepsis. Am J Respir Crit Care Med..

[22] Gauer R, Forbes D, Boyer N (2020). Sepsis: Diagnosis and Management. Am Fam Physician..

[23] Berg D, Gerlach H (2018) Recent advances in understanding and managing sepsis. F1000Res. 7:F1000 Faculty Rev-1570. 10.12688/f1000research.15758.1 10.12688/f1000research.15758.1 PMC6173111 30345006

[24] Shankar-Hari M, Phillips GS, Levy ML (2016). Developing a New Definition and Assessing New Clinical Criteria for Septic Shock: For the Third International Consensus Definitions for Sepsis and Septic Shock (Sepsis-3). JAMA..

[25] Bateman RM, Sharpe MD, Jagger JE (2016). 36th International Symposium on Intensive Care and Emergency Medicine: Brussels, Belgium. 15-18 March 2016. Crit Care.

[26] Pierrakos C, Velissaris D, Bisdorff M, Marshall JC, Vincent JL (2020). Biomarkers of sepsis: time for a reappraisal. Crit Care..

[27] Faix JD (2013). Biomarkers of sepsis. Crit Rev Clin Lab Sci..

