# Supplementary material for: Interdisciplinary perspectives on computed tomography in sepsis: survey among medical doctors at a large university medical center
Source: Eur Radiol. 2023 Jul 14;33(12):9296–308. doi: 10.1007/s00330-023-09842-3 (PMC10667150; doi:10.1007/s00330-023-09842-3)
Supplement: Supplementary file 1 — (PDF 413 kb) [file 330_2023_9842_MOESM1_ESM.pdf]

		**Work experience in years** n (%)				
		<3 years (n=74/371, 19.9%)	>3-≤7 years (n=130/371, 35.0%)	>7-≤11 years (n=73/371), 19.7%)	>11-≤20 years (n=64/371, 17.3%)	>20 years (n=30/371, 8.1%)
**Position**	Assistant physician (n=191/371, 51.5%)	74 (38.7)	109 (57.1)	6 (3.1)	1 (0.5)	1 (0.5)
	Board-certified physician (n=99/371, 26.7%)	0 (0.0)	20 (20.2)	54 (54.5)	20 (20.2)	5 (5.1)
	Senior or chief physician (n=81/371, 21.8%)	0 (0.0)	1 (1.2)	13 (16.0)	43 (53.1)	24 (29.6)

Table S1: Overview of the position and work experience of participants with line-related percentages.

The higher the position, the more work experience physicians tend to have. The group with <3 years of work experience only consists of assistant physicians. The assistant physician group consists mainly of participants with >3-≤7 years (57.1%, n=109/191) of experience, followed by <3 years (38.7%, n=74/191) of experience. While most board-certified physicians stated a work experience of >7-≤11 years (54.5%, n=54/99), senior or chief physicians had worked for >11-≤20 years (53.1%, n=43/81).

		Workplace n (%)						
		ICU n=115/371	General ward n=82/371	Emergency department n=50/371	OR n=42/371	Radiology department n=32/371	Outpatient clinic n=32/371	Other n=18/371
Position	Assistant physician (n=191/371, 51.5%)	47 (24.6)	60 (31.4)	33 (17.3)	20 (10.5)	15 (7.9)	6 (3.1)	10 (5.2)
	Board-certified physician (n=99/371, 26.7%)	39 (39.4)	13 (13.1)	8 (8.1)	10 (10.1)	9 (9.1)	13 (13.1)	7 (7.1)
	Senior or chief physician (n=81/371, 21.8%)	29 (35.8)	9 (11.1)	9 (11.1)	12 (14.8)	8 (9.9)	13 (16.0)	1 (1.2)
Work experience	<3 years (n=74/371, 19.9%)	13 (17.6)	31 (41.9)	15 (20.3)	6 (8.1)	5 (6.8)	2 (2.7)	2 (2.7)
	>3-≤7 years (n=130/371, 35.0%)	38 (29.2)	33 (25.4)	17 (13.1)	14 (10.8)	14 (10.8)	7 (5.4)	7 (5.4)
	>7-≤11 years (n=73/371), 19.7%)	34 (46.6)	8 (11.0)	5 (6.8)	8 (11.0)	6 (8.2)	7 (9.6)	5 (6.8)
	>11-≤20 years (n=64/371, 17.3%)	24 (37.5)	7 (10.9)	8 (12.5)	11 (17.2)	5 (7.8)	7 (10.9)	2 (3.1)
	>20 years (n=30/371, 8.1%)	6 (20.0)	3(10.0)	5 (16.7)	3 (10.0)	2 (6.7)	9 (30.0)	2 (6.7)

Table S2: Distribution patterns of the different positions and work experiences related to the participants' workplace with line-related percentages.

Most board-certified and senior or chief physicians from our study population work at an ICU (39.4%, n=39/99; 35.8%, n=29/81, respectively). Otherwise, both positions are quite homogeneously distributed among the different workplaces. Assistant physicians were least likely to work in the outpatient department (3.1%, n=6/191). The majority of physicians with >20 years of work experience reported working in the outpatient department or ICU. While physicians with <3 years of experience were most likely to cite general wards as their workplace, it was the ICU for

physicians with >3-≤7 or >7-≤11 years of experience. Radiology and emergency departments were most represented by assistant physicians and physicians with >3-≤7 years of work experience.

ICU = Intensive care unit; OR = Operation room

Medical specialty		Workplace n (%)						
		ICU n=114/370	General ward n=82/370	Emergency department n=50/370	OR n=42/370	Radiology department n=32/370	Outpatient clinic n=32/370	Other n=18/370
Medical specialty	Internal medicine physicians (n=157/370, 42.4%)	51 (32.5)	47 (29.9)	38 (24.2)	0 (0.0)	0 (0.0)	16 (10.2)	5 (3.2)
	Surgeons (n=44/370, 11.9%)	4 (9.1)	14 (31.8)	3 (6.8)	19 (43.2)	0 (0.0)	2 (4.5)	2 (4.5)
	Anesthesiologists (n=70/370, 18.9%)	44 (62.9)	1 (1.4)	1 (1.4)	21 (30.0)	0 (0.0)	2 (2.9)	1 (1.4)
	Radiologists (n=33/370, 8.9%)	0 (0.0)	0 (0.0)	0 (0.0)	0 (0.0)	32 (97.0)	0 (0.0)	1 (3.0)
	Other medical specialties (n=66/370, 17.8%)	15 (22.7)	20 (30.3)	8 (12.1)	2 (3.0)	0 (0.0)	12 (18.2)	9 (13.6)

Table S3 Overview of survey participants according to workplace and medical specialty with line-related percentages.

Most internal medicine physicians worked in the ICU (32.5%, n=51/157) and in general wards (29.9%, n=47/157), followed by the emergency department (24.2%, n=38/157). In the latter, they made up the largest proportion of physicians at 76.0% (n=38/50). The surgeons' workplaces were mainly distributed between the OR (43.2%, n=19/44) and the general ward (31.8%, n=14/44). Anesthesiologists accounted for the second largest percentage of physicians in ICUs (38.6%, n=44/114). Of the "other medical specialty group", most reported working at general wards (30.3%, n=20/66) or ICU (22.7%, n=15/66).

ICU = Intensive care unit; OR = Operation room

		Do you deal with septic patients in your daily clinical routine?	
		YES<br>n (%)	NO<br>n (%)
**Medical specialty** (n=370)	Internal medicine physicians (n=157/370, 42.4%)	149 (94.9)	8 (5.1)
	Surgeons (n=44/370, 11.9%)	42 (95.5)	2 (4.5)
	Anesthesiologists (n=70/370, 18.9%)	69 (98.6)	1 (1.4)
	Radiologists (n=33/370, 8.9%)	31 (93.3)	2 (6.1)
	Other medical specialties (n=66/370, 17.8%)	54 (81.8)	12 (18.2)

Table S4: Overview of physicians' involvement in managing septic patients in their daily clinical routine listed by the medical specialty.

The majority of participants indicated to deal with septic patients in their daily clinical routine. In each medical specialty, notably more physicians reported being involved in the management of patients with sepsis than did not. The "other medical specialty" group was the most likely to state not dealing with septic patients on a daily basis (18.2%, n=12/66).

		Work experience in years (n=371)																			
		<3 (n=74)				>3-≤7 (n=130)				>7-≤11 (n=73)				>11-≤20 (n=64)				>20 (n=30)			
		1	2	3	4	1	2	3	4	1	2	3	4	1	2	3	4	1	2	3	4
The following clinical criteria speak for a CT scan:	SOFA	2.7 (2)	12.2 (9)	50.0 (37)	35.1 (26)	7.7 (10)	10.0 (13)	53.1 (69)	29.2 (38)	5.5 (4)	13.7 (10)	57.5 (42)	23.3 (17)	9.4 (6)	15.6 (10)	59.4 (38)	15.6 (10)	16.7 (5)	16.7 (5)	43.3 (13)	23.3 (7)
	qSOFA	6.8 (5)	10.8 (8)	48.6 (36)	33.8 (25)	4.6 (6)	11.5 (15)	53.8 (70)	30.0 (39)	4.1 (3)	21.9 (16)	53.4 (39)	20.5 (15)	12.5 (8)	23.4 (15)	45.3 (29)	18.8 (12)	13.3 (4)	20.0 (6)	36.7 (11)	30.0 (9)
	Fever or hypothermia	5.4 (4)	32.4 (24)	44.6 (33)	17.6 (13)	8.5 (11)	20.8 (27)	53.8 (70)	16.9 (22)	8.2 (6)	27.4 (29)	49.3 (36)	15.1 (11)	9.4 (6)	18.8 (12)	53.1 (34)	18.8 (12)	6.7 (2)	16.7 (5)	53.3 (16)	23.3 (7)
	SIRS criteria	5.4 (4)	23.0 (17)	55.4 (41)	16.2 (12)	9.2 (12)	14.6 (19)	57.7 (75)	18.5 (24)	5.5 (4)	32.9 (24)	49.3 (36)	12.3 (9)	6.3 (4)	18.8 (12)	57.8 (37)	17.2 (11)	13.3 (4)	13.3 (4)	56.7 (17)	16.7 (5)
	RR ≥ 22/min	6.8 (5)	25.7 (19)	56.8 (42)	10.8 (8)	7.7 (10)	32.3 (42)	46.9 (61)	13.1 (17)	8.2 (6)	47.9 (35)	35.6 (26)	8.2 (6)	12.5 (8)	35.9 (23)	39.1 (25)	12.5 (8)	13.3 (4)	16.7 (5)	50.0 (15)	20.0 (6)
	Postoperative patient	10.8 (8)	21.6 (16)	50.0 837)	17.6 (13)	15.4 (20)	17.7 (23)	47.7 (62)	19.2 (25)	9.6 (7)	28.8 (21)	49.3 (36)	12.3 (9)	7.8 (5)	25.0 (16)	40.6 (26)	26.6 (17)	20.0 (6)	10.0 (3)	50.0 (15)	20.0 (6)
	Increased catecholamine demand	1.4 (1)	16.2 (12)	54.1 (40)	28.4 (21)	4.6 (6)	10.8 (14)	53.1 (69)	31.5 (41)	1.4 (1)	11.0 (8)	54.8 (40)	32.9 (24)	4.7 (3)	7.8 (5)	51.6 (33)	35.9 (23)	13.3 (4)	10.0 (3)	43.3 (13)	33.3 (10)
	Signs of reduced vigilance	1.4 (1)	6.8 (5)	45.9 (34)	45.9 (34)	4.6 (6)	9.2 (12)	46.9 (61)	39.2 (51)	0.0 (0)	9.6 (7)	50.7 (37)	39.7 (29)	3.1 (2)	3.1 (2)	43.8 (28)	50.0 (32)	10.0 (3)	6.7 (2)	43.3 (13)	40.0 (12)
	Immunosuppression	2.7 (2)	33.8 (25)	51.4 (38)	12.2 (9)	6.9 (9)	22.3 (29)	52.3 (68)	18.5 (24)	5.5 (4)	19.2 (14)	54.8 (40)	20.5 (15)	1.6 (1)	17.2 (11)	51.6 (33)	29.7 (19)	3.3 (1)	10.0 (3)	66.7 (20)	20.0 (6)
	SBP < 100 or MAP < 65 – 70 mmHg	4.1 (3)	27.0 (20)	45.9 (34)	23.0 (17)	5.4 (7)	23.1 (30)	50.0 (65)	21.5 (28)	5.5 (4)	24.7 (18)	57.5 (42)	12.3 (9)	3.1 (2)	15.6 (10)	57.8 (37)	23.4 (15)	13.3 (4)	13.3 (4)	46.7 (14)	26.7 (8)
	Elderly patient	10.8 (8)	50.0 (37)	37.8 (28)	1.4 (1)	16.9 (22)	43.8 (57)	31.5 (41)	7.7 (10)	13.7 (10)	53.4 (39)	27.4 (20)	5.5 (4)	14.1 (9)	46.9 (30)	29.7 (19)	9.4 (6)	26.7 (8)	36.7 (11)	23.3 (7)	13.3 (4)
The following ancillary parameter speak for a CT:	Elevated PCT	6.8 (5)	10.8 (8)	51.4 (38)	31.1 (23)	3.1 (4)	8.5 (11)	59.2 (77)	29.2 (38)	6.8 (5)	5.5 (4)	65.8 (48)	21.9 (16)	4.7 (3)	21.9 (14)	43.8 (28)	29.7 (19)	6.7 (2)	26.7 (2)	36.7 (11)	30.0 (9)
	Elevated CRP	4.1 (3)	25.7 (19)	56.8 (42)	13.5 (10)	3.8 (5)	22.3 (29)	56.2 (73)	17.7 (23)	9.6 (7)	20.5 (15)	52.1 (38)	17.8 (13)	7.8 (5)	25.0 (16)	40.6 (26)	26.6 (17)	6.7 (2)	23.3 (7)	50.0 (15)	20.0 (6)
	Leukocytosis or leukopenia	6.8 (5)	20.3 (15)	56.8 (42)	16.2 (12)	2.3 (3)	16.9 (22)	59.2 (77)	21.5 (28)	6.8 (5)	17.8 (13)	60.3 (44)	15.1 (11)	3.1 (2)	21.9 (14)	45.3 (29)	29.7 (19)	6.7 (2)	23.3 (7)	56.7 (17)	13.3 (4)
	Elevated IL-6	17.6 (13)	44.6 (33)	32.4 (24)	5.4 (4)	16.9 (22)	40.8 (53)	34.6 (45)	7.7 (10)	17.8 (13)	35.6 (26)	41.1 (30)	5.5 (4)	25.0 (16)	34.4 (22)	32.8 (21)	7.8 (5)	10.0 (3)	46.7 (14)	36.7 (11)	6.7 (2)
	Elevated lactate levels	1.4 (1)	16.2 (12)	48.6 (36)	33.8 (25)	3.1 (4)	11.5 (15)	61.5 (80)	23.8 (31)	4.1 (3)	13.7 (10)	58.9 (43)	23.3 (17)	3.1 (2)	10.9 (7)	53.1 (34)	32.8 (21)	6.7 (2)	16.7 (5)	46.7 (14)	30.0 (9)
	Sonographically suspected infection focus	4.1 (3)	10.8 (8)	33.8 (25)	51.4 (38)	2.3 (3)	15.4 (20)	43.8 (57)	38.5 (50)	1.4 (1)	20.5 (15)	41.1 (30)	37.0 (27)	3.1 (2)	12.5 (8)	29.7 (19)	54.7 (35)	3.3 (1)	3.3 (1)	50.0 (15)	43.3 (13)
	Abnormal chest x- ray	5.4 (4)	36.5 (27)	28.4 (21)	29.7 (22)	5.4 (7)	32.3 (42)	43.8 (57)	18.5 (24)	1.4 (1)	35.6 (26)	41.1 (30)	21.9 (16)	3.1 (2)	21.9 (14)	45.3 (29)	29.7 (19)	16.7 (5)	6.7 (2)	43.3 (13)	33.3 (10)
	SARS-CoV-2 detected	8.1 (6)	24.3 (18)	50.0 (37)	17.6 (13)	6.2 (8)	19.2 (25)	48.5 (63)	26.2 (34)	1.4 (1)	34.2 (25)	43.8 (32)	20.5 (15)	3.1 (2)	12.5 (8)	54.7 (35)	29.7 (19)	3.3 (1)	30.0 (9)	40.0 (12)	26.7 (8)
	Positive blood culture	5.4 (4)	33.8 (25)	50.0 (37)	10.8 (8)	2.3 (3)	33.1 (43)	41.5 (54)	23.1 (30)	2.7 (2)	35.6 (26)	49.3 (36)	12.3 (9)	6.3 (4)	15.6 (10)	53.1 (34)	25.0 (16)	6.7 82)	16.7 (5)	53.3 (16)	23.3 (7)

Eur Radiol (2023) Opper Hernando MI, Witham D, Steinhagen PR et al

Table S5 Absolute and relative frequencies in the analysis of clinical and ancillary criteria in relation to work experience in years.

No statistically significant differences in responses for the clinical criteria were found between physicians grouped by years of work experience. For elevated PCT, the tendency to choose this criterion as an indication for a CT examination in sepsis decreased with years of experience of the respondent (p=0.025). While agreement with this criterion was high among physicians with >3-≤7 years at 88.4% (n=115/130), only 66.7% (n=20/30) of physicians with >20 years of experience still considered PCT relevant for requesting a CT. For abnormal chest X-ray findings, years of experience also played a role (p=0.007), but an opposite trend was apparent: only 58.1% (n=43/74) of physicians with <3 years of experience but 76.6% (n=23/30) of physicians with >20 years of experience considered this a relevant criterion for a CT in sepsis.

1= strongly disagree; 2= somewhat disagree; 3= somewhat agree; 4= strongly agree

CT = Computed tomography; SOFA = Systemic Organ Failure Assessment; qSOFA = Quick Systemic Organ Failure Assessment; SIRS = Systemic Inflammatory Response Syndrome; RR = Respiratory rate; SBP = Systolic blood pressure; MAP = Mean arterial pressure; PCT = Procalcitonin; CRP = C-reactive protein; IL-6 = Interleukin-6; SARS-CoV-2 = Severe acute respiratory syndrome coronavirus type 2

		Workplace (n=371)																											
		ICU (n=115)				General ward (n=82)				Emergency department (n=50)				OR (n=42)				Radiology (n=32)				Outpatient clinic (n=32)				Other (n=18)			
		1	2	3	4	1	2	3	4	1	2	3	4	1	2	3	4	1	2	3	4	1	2	3	4	1	2	3	4
The following clinical criteria speak for a CT scan:	SOFA	9.6 (11)	13.9 (16)	54.8 (63)	21.7 (25)	6.1 (5)	12.2 (10)	50.0 (41)	31.7 (26)	8.0 (4)	10.0 (5)	62.0 (31)	20.0 (10)	4.8 (2)	16.7 (7)	52.4 (22)	26.2 (11)	3.1 (1)	9.4 (3)	50.0 (16)	37.5 (12)	9.4 (3)	18.8 (6)	50.0 (16)	21.9 (7)	5.6 (1)	0.0 (0)	55.6 (10)	38.9 (7)
	qSOFA	11.3 (13)	21.7 (25)	51.3 (59)	15.7 (18)	3.7 (3)	11.0 (9)	50.0 (41)	35.4 (29)	4.0 (2)	12.0 (6)	56.0 (28)	28.0 (14)	9.5 (4)	14.3 (6)	52.4 (22)	23.8 (10)	3.1 (1)	12.5 (4)	50.0 (16)	34.4 (11)	6.3 (2)	21.9 (7)	40.6 (13)	31.3 (10)	5.6 (1)	16.7 (3)	33.3 (6)	44.4 (8)
	Fever or hypothermia	8.7 (10)	30.4 (35)	48.7 (56)	12.2 (14)	8.5 (7)	26.8 (22)	50.0 (41)	14.6 (12)	16.0 (8)	16.0 (8)	58.0 (29)	10.0 (5)	0.0 (0)	19.0 (8)	57.1 (24)	23.8 (10)	0.0 (0)	3.1 (1)	62.5 (20)	34.4 (11)	9.4 (3)	31.3 (10)	34.4 (11)	25.0 (8)	5.6 (1)	22.2 (4)	44.4 (8)	27.8 (5)
	SIRS criteria	12.2 (14)	26.1 (30)	52.2 (60)	9.6 (11)	6.1 (5)	23.2 (19)	52.4 (43)	18.3 (15)	6.0 (3)	20.0 (10)	64.0 (32)	10.0 (5)	4.8 (2)	16.7 (7)	59.5 (25)	19.0 (8)	0.0 (0)	6.3 (2)	68.8 (22)	25.0 (8)	6.3 (2)	18.8 (6)	50.0 (16)	25.0 88)	11.1 (2)	11.1 (2)	44.4 (8)	33.3 (6)
	RR ≥ 22/min	9.6 (11)	45.2 (52)	34.8 840)	10.4 (12)	7.3 (6)	26.8 (22)	54.9 (45)	11.0 (9)	8.0 (4)	30.0 (15)	54.0 (27)	8.0 (4)	9.5 (4)	38.1 (16)	45.2 (19)	7.1 (3)	6.3 (2)	25.0 (8)	50.0 (16)	18.8 (6)	12.5 (4)	25.0 (8)	37.5 (12)	25.0 (8)	11.1 82)	16.7 (3)	55.6 (10)	16.7 (3)
	Postoperative patient	14.8 (17)	26.1 (30)	44.3 (51)	14.8 (17)	11.0 (9)	15.9 (13)	47.6 (39)	25.6 (21)	8.0 (4)	26.0 (13)	44.0 (22)	22.0 (11)	7.1 (3)	23.8 (10)	54.8 (23)	14.3 (6)	12.5 (4)	18.8 (6)	46.9 (15)	21.9 (7)	12.5 (4)	12.5 (4)	53.1 (17)	21.9 (7)	27.8 (5)	16.7 (3)	50.0 (9)	5.6 (1)
	Increased catecholamine demand	3.5 (4)	10.4 (12)	53.0 (61)	33.0 (38)	4.9 (4)	9.8 (8)	56.1 (46)	29.3 (24)	6.0 (3)	12.0 (6)	48.0 (24)	34.0 (17)	2.4 (1)	7.1 (3)	54.8 (23)	35.7 (15)	0.0 (0)	12.5 (4)	40.6 (13)	46.9 (15)	6.3 (2)	18.8 (6)	56.3 (18)	18.8 86)	5.6 (1)	16.7 (3)	55.6 (10)	22.2 (4)
	Signs of reduced vigilance	3.5 (4)	4.3 (5)	46.1 (53)	46.1 (53)	3.7 (3)	6.1 (5)	52.4 (43	37.8 (31)	4.0 (2)	10.0 (5)	42.0 (21)	44.0 (22)	0.0 (0)	14.3 (6)	38.1 (16)	47.6 (20)	3.1 (1)	12.5 (4)	46.9 (15)	37.5 (12	3.1 (1)	9.4 (3)	46.9 (15)	40.6 (13)	5.6 (1)	0.0 (0)	55.6 (10)	38.9 (7)
	Immunosuppression	6.1 (7)	17.4 (20)	53.0 (61)	23.5 (27)	3.7 83)	28.0 (23)	51.2 (42)	17.1 (14)	2.0 (1)	20.0 (10)	66.0 (33)	12.0 (6)	2.4 (1)	38.1 (16)	47.6 (20)	11.9 (5)	6.3 (2)	21.9 87)	40.6 813)	31.3 (10)	3.1 (1)	6.3 (2)	65.6 (21)	25.0 (8)	11.1 82)	22.2 (4)	50.0 (9)	16.7 (3)
	SBP < 100 or MAP < 65 – 70 mmHg	8.7 (10)	20.9 (24)	53.0 (61)	17.4 (20)	2.4 (2)	20.7 (17)	56.1 (46)	20.7 (17)	4.0 (2)	26.0 (13)	44.0 (22)	26.0 (13)	2.4 (1)	33.3 (14)	50.0 (21)	14.3 (6)	0.0 (0)	15.6 (5)	50.0 (16)	34.4 (11)	3.1 (1)	15.6 (5)	59.4 (19)	21.9 (7)	22.2 (4)	22.2 (4)	38.9 (7)	16.7 (3)
	Elderly patient	15.7 (18)	58.3 (67)	20.9 (24)	5.2 (6)	15.9 (13)	47.6 (39)	32.9 (27)	3.7 (3)	14.0 (7)	32.0 (16)	48.0 (24)	6.0 (3)	16.7 (7)	40.5 (17)	33.3 (14)	9.5 (4)	9.4 (3)	40.6 (13)	34.4 (11)	15.6 (5)	9.4 (3)	53.1 (17)	28.1 (9)	9.4 (3)	33.3 (6)	27.8 (5)	33.3 (6)	5.6 (1)
The following ancillary parameter speak for a CT:	Elevated PCT	1.7 (2)	11.3 (13)	60.9 (70)	26.1 (30)	7.3 (6)	8.5 (7)	54.9 (45)	29.3 (24)	6.0 (3)	22.0 (11)	44.0 (22)	28.0 (14)	2.4 (1)	7.1 (3)	59.5 (25)	31.0 (13)	6.3 (2)	6.3 (2)	53.1 (17)	34.4 (11)	9.4 (3)	28.1 (9)	40.6 (13)	21.9 (7)	11.1 (2)	0.0 (0)	55.6 (10)	33.3 (6)
	Elevated CRP	4.3 (5)	30.4 (35)	52.2 (60)	13.0 (15)	7.3 (6)	14.6 (12)	58.5 (48)	19.5 (16)	6.0 (3)	30.0 (15)	56.0 (28)	8.0 (4)	7.1 (3)	19.0 (8)	40.5 (17)	33.3 (14)	0.0 (0)	12.5 (4)	53.1 (17)	34.4 (11)	12.5 (4)	31.3 (19)	40.6 (13)	15.6 (5)	5.6 (1)	11.1 (2)	61.1 (11)	22.2 (4)
	Leukocytosis or leukopenia	1.7 (2)	25.2 (29)	54.8 (63)	18.3 (21)	7.3 (6)	15.9 (13)	57.3 (47)	19.5 (16)	6.0 (3)	24.0 (12)	62.0 (31)	8.0 (4)	7.1 (3)	11.9 (5)	50.0 (21)	31.0 (13)	0.0 (0)	3.1 (1)	62.5 (20)	34.4 (11)	3.1 (1)	28.1 (9)	50.0 (16)	18.8 (6)	11.1 (2)	11.1 (2)	61.1 (11)	16.7 (3)
	Elevated IL-6	19.1 (2)	40.0 (46)	31.3 (36)	9.6 (11)	22.0 (18)	42.7 (35)	28.0 (23)	7.3 (6)	18.0 (9)	36.0 (18)	42.0 (21)	4.0 (2)	16.7 (7)	45.2 (19)	28.6 (12)	9.5 (4)	9.4 (3)	28.1 (9)	62.5 (20)	0.0 (0)	15.6 (5)	50.0 (16)	31.3 (10)	3.1 (1)	16.7 (3)	27.8 (5)	50.0 (9)	5.6 (1)
	Elevated lactate levels	1.7 (2)	11.3 (13)	51.3 (59)	35.7 (41)	1.2 (1)	13.4 (11)	54.9 (45)	30.5 (25)	8.0 (4)	6.0 (3)	66.0 (33)	20.0 (10)	2.4 (1)	11.9 (5)	73.8 (31)	11.9 (5)	0.0 (0)	9.4 (3)	50.0 (16)	40.6 (13)	3.1 (1)	31.3 (10)	50.0 (16)	15.6 (5)	16.7 (3)	22.2 (4)	38.9 (7)	22.2 (4)
	Sonographically suspected infection focus	2.6 (3)	13.0 (15)	43.5 (50)	40.9 (47)	1.2 (1)	14.6 (12)	39.0 (32)	45.1 (37)	4.0 (2)	16.0 (8)	40.0 (20)	40.0 (20)	2.4 (1)	11.9 (5)	23.8 (10)	61.9 (26)	3.1 (1)	18.8 (6)	43.8 (14)	34.4 (11)	0.0 (0)	12.5 (4)	46.9 (15)	40.6 (13)	11.1 (2)	11.1 (2)	27.8 (5)	50.0 (9)
	Abnormal chest x-ray	2.6 (3)	27.8 (32)	46.1 (53)	23.5 (27)	2.4 (2)	42.7 (35)	28.0 (23)	26.8 (22)	12.0 (6)	36.0 (18)	32.0 (16)	20.0 (10)	4.8 (2)	23.8 (10)	38.1 (16)	33.3 (14)	9.4 (3)	21.9 (7)	50.0 (16)	18.8 (6)	3.1 (1)	12.5 (4)	50.0 (16)	34.4 (11)	11.1 (2)	27.8 (5)	55.6 (10)	5.6 (1)
	SARS-CoV-2 detected	4.3 (5)	17.4 (20)	43.5 (50)	34.8 (40)	7.3 (6)	28.0 (23)	51.2 (42)	13.4 (11)	4.0 (2)	28.0 (14)	50.0 (25)	18.0 (9)	2.4 (1)	28.6 (12)	42.9 (18)	26.2 (11)	6.3 (2)	18.8 (6)	62.5 (20)	12.5 (4)	0.0 (0)	18.8 (6)	56.3 (18)	25.0 (8)	11.1 (2)	22.2 (4)	33.3 (6)	33.3 (6)
	Positive blood culture	3.5 (4)	30.4 (35)	46.1 (53)	20.0 (23)	1.2 (1)	32.9 (27)	51.2 (42)	14.6 (12)	8.0 (4)	44.0 (22)	42.0 (21)	6.0 (3)	2.4 (1)	31.0 (13)	42.9 (18)	23.8 (10)	3.1 (1)	9.4 (3)	53.1 (17)	34.4 (11)	9.4 (3)	25.0 (8)	43.8 (14)	21.9 (7)	5.6 (1)	5.6 (1)	66.7 (12)	22.2 (4)

<u>**Table S6** Absolute and relative frequencies in the analysis of clinical and ancillary criteria in relation to workplace.</u>

Compared with other workplaces, significantly more radiologists (96.9%, n=31/32) classified “fever or hypothermia” as a clinical parameter supporting CT in patients with sepsis (p=0.005).

While only 12.5% (n=4/33) of radiologists rejected elevated CRP levels as an indication for CT in sepsis, significantly more physicians working in the ICU (34.7%, n=40/115), emergency department (36.0%, n=18/50), and outpatient clinic (43.8%, n=14/32) disagreed (p=0.019). Significant differences were also seen in responses between various workplaces regarding "leukocytosis or leukopenia" (p=0.047). At 96.9% (n=31/32), most radiologists considered leukocytosis or leukopenia a CT indication. Conversely, significantly fewer physicians working in the emergency department (70.0%, n=35/50) and outpatient clinic (68.8%, n=22/32) considered this criterion relevant. With percentages ranging from 85.4% (n=70/82, general ward) to 90.6% (n=29/32, radiology), at least 20% more participants considered elevated lactate levels as an indication for a CT examination in patients with sepsis compared to physicians from outpatient clinic (65.6%, n=21/32) or other workplaces (61.1%, n=11/18) (p<0.001). Significant differences were also observed for abnormal chest X-ray, significant differences in responses were found concerning the workplace (p=0.015). Physicians in the general ward (54.8%, n=45/82) and emergency department (52.0%, n=26/50) were least likely to agree that an abnormal X-ray is a CT indication in septic patients. While 87.5% (n=28/32) of radiologists considered a positive blood culture as decisive for a CT request, only 48.0% (n=24/50) of emergency department physicians did (p=0.028).

1= strongly disagree; 2= somewhat disagree; 3= somewhat agree; 4= strongly agree

CT = Computed tomography; SOFA = Systemic Organ Failure Assessment; qSOFA = Quick Systemic Organ Failure Assessment; SIRS = Systemic Inflammatory Response Syndrome; RR = Respiratory rate; SBP = Systolic blood pressure; MAP= Mean arterial pressure; PCT = Procalcitonin; CRP = C-reactive protein; IL-6 = Interleukin-6; SARS-CoV-2 = Severe acute respiratory syndrome coronavirus type 2; ICU = Intensive care unit; OR = Operation room

		Medical specialty (n=370)																			
		Internal medicine (n=157)				Surgery (n=44)				Radiology (n=33)				Anesthesiology (n=70)				Other (n=66)			
		1	2	3	4	1	2	3	4	1	2	3	4	1	2	3	4	1	2	3	4
The following clinical criteria speak for a CT scan:	SOFA	8.9 (14)	12.7 (20)	54.8 (86)	23.6 (37)	11.4 (5)	13.6 (6)	40.9 (18)	34.1 (15)	3.0 (1)	9.1 (3)	51.5 (17)	36.4 (12)	4.3 (3)	11.4 (8)	62.9 (44)	21.4 (15)	6.1 (4)	13.6 (9)	51.5 (34)	28.8 (19)
	qSOFA	5.7 (9)	17.2 (27)	51.0 (80)	26.1 (41)	11.4 (5)	11.4 (5)	45.5 (20)	31.8 (14)	3.0 (1)	12.1 (4)	51.5 (17)	33.3 (11)	10.0 (7)	20.0 (14)	51.4 (36)	18.6 (13)	6.1 (4)	15.2 (10)	47.0 (31)	31.8 (21)
	Fever or hypothermia	14.0 (22)	28.7 (45)	44.6 (70)	12.7 (20)	2.3 (1)	22.7 (10)	54.5 (24)	20.5 (9)	0.0 (0)	3.1 (1)	63.6 (21)	33.3 (11)	4.3 (3)	27.1 (19)	55.7 (39)	12.9 (9)	4.5 (3)	19.7 (13)	51.5 (34)	24.2 (16)
	SIRS criteria	8.9 (14)	23.6 (37)	52.9 (83)	14.6 (23)	6.8 (3)	15.9 (7)	61.4 (27)	15.9 (7)	0.0 (0)	6.1 (2)	69.7 (23)	24.2 (8)	11.4 (8)	22.9 (16)	55.7 (39)	10.0 (7)	4.5 (3)	21.2 (14)	50.0 (33)	24.2 (16)
	RR ≥ 22/min	8.3 (13)	33.1 (52)	46.5 (73)	12.1 (19)	11.4 (5)	25.0 (11)	59.1 (26)	4.5 (2)	6.1 (2)	24.2 (8)	51.5 (17)	18.2 (6)	11.4 (8)	44.3 (31)	34.3 (24)	10.0 (7)	7.6 (5)	31.8 (21)	43.9 (29)	16.7 (11)
	Postoperative patient	11.5 (18)	17.8 (28)	54.1 (85)	16.6 (26)	6.8 (3)	13.6 (6)	45.5 (20)	34.1 (15)	12.1 (4)	18.2 (6)	48,6 (16)	21.2 (7)	15.7 (11)	34.3 (24)	42.9 (30)	7.1 (5)	15.2 (10)	22.7 (15)	37.9 (25)	24.2 (16)
	Increased catecholamine demand	5.1 (8)	12.1 (19)	53.5 (84)	29.3 (46)	4.5 (2)	4.5 (2)	56.8 (25)	34.1 (15)	0.0 (0)	12.1 (4)	42.4 (14)	45.5 (15)	2.9 (2)	8.6 (6)	55.7 (39)	32.9 (23)	4.5 (3)	16.7 (11)	50.0 (33)	28.8 (19)
	Signs of reduced vigilance	4.5 (7)	7.0 (11)	48.4 (76)	40.1 (63)	4.5 (2)	9.1 (4)	45.5 (20)	40.9 (18)	3.0 (1)	12.1 (4)	48.5 (16)	36.4 (12)	1.4 (1)	7.1 (5)	42.9 (30)	48.6 (34)	1.5 (1)	6.1 (4)	47.0 (31)	45.5 (30)
	Immunosuppression	4.5 (7)	15.9 (25)	58.6 (92)	21.0 (33)	4.5 (2)	31.8 (14)	50.0 (22)	13.6 (6)	6.1 (2)	21.2 (7)	42.4 (14)	30.3 (10)	7.1 (5)	35.7 (25)	38.6 (27)	18.6 (13)	1.5 (1)	16.7 (11)	66.7 (44)	15.2 (10)
	SBP < 100 or MAP < 65 – 70 mmHg	7.0 (11)	22.9 (36)	49.7 (78)	20.4 (32)	2.3 (1)	15.9 (7)	61.4 (27)	20.5 (9)	0.0 (0)	15.2 (5)	51.5 (17)	33.3 (11)	5.7 (4)	28.6 (20)	50.0 (35)	15.7 (11)	6.1 (4)	21.2 814)	51.5 (34)	21.2 (14)
	Elderly patient	14.6 (23)	48.4 (76)	29.9 847)	7.0 (11)	11.4 (5)	45.5 (20)	36.4 (16)	6.8 (3)	9.1 (3)	39.4 (13)	36.4 (12)	15.2 (5)	17.1 (12)	60.0 (42)	17.1 (12)	5.7 (4)	21.2 (14)	34.8 (23)	40.9 (27)	3.0 (2)
The following ancillary parameter speak for a CT:	Elevated PCT	7.6 (12)	14.6 (23)	52.2 (82)	25.5 (40)	0.0 (0)	6.8 (3)	54.5 (24)	38.6 (17)	6.1 (2)	6.1 (2)	54.5 (18)	33.3 (11)	1.4 (1)	7.1 (5)	68.6 (48)	22.9 (16)	6.1 (4)	18.2 (12)	43.9 (29)	31.8 (21)
	Elevated CRP	8.3 (13)	24.2 (38)	54.1 (85)	13.4 (21)	2.3 (1)	4.5 82)	52.3 (23)	40.9 (18)	0.0 (0)	12.1 (4)	51.5 (17)	36.4 (12)	7.1 (5)	32.9 (23)	50.0 (35)	10.0 (7)	4.5 (3)	27.3 (18)	51.5 (34)	16.7 (11)
	Leukocytosis or leukopenia	6.4 (10)	26.1 (41)	53.5 (84)	14.0 (22)	4.5 (2)	2.3 (1)	56.8 (25)	36.4 (16)	0.0 (0)	3.0 (1)	63.6 (21)	33.3 (11)	2.9 (2)	20.0 (14)	61.4 (43)	15.7 (11)	4.5 (3)	19.7 (13)	54.5 (36)	21.2 (14)
	Elevated IL-6	21.7 (34)	42.0 (66)	31.2 (49)	5.1 (8)	22.7 (10=	52.3 (23)	18.2 (8)	6.8 (3)	9.1 (3)	27.3 (9)	63.6 (21)	0.0 (0)	15.7 (11)	30.0 (21)	42.9 (30)	11.4 (8)	13.6 (9)	43.9 (29)	33.3 (22)	9.1 (6)
	Elevated lactate levels	3.8 (6)	13.4 (21)	56.1 (88)	26.8 (42)	2.3 (1)	18.2 (8)	56.8 (25)	22.7 (10)	0.0 (0)	9.1 (3)	48.5 (16)	42.4 (14)	2.9 (2)	7.1 (5)	58.6 (41)	31.4 (22)	4.5 (3)	16.7 (11)	56.1 (37)	22.7 (15)
	Sonographically suspected infection focus	1.9 (3)	14.0 (22)	45.2 (71)	38.9 (61)	2.3 (1)	13.6 (6)	31.8 (14)	52.3 (23)	3.0 (1)	18.2 (6)	45.5 (15)	33.3 (11)	1.4 (1)	12.9 (9)	32.9 (23)	52.9 (37)	6.1 (4)	13.6 (9)	34.8 (23)	45.5 (30)
	Abnormal chest x- ray	5.7 (9)	30.6 (48)	40.8 (64)	22.9 (36)	2.3 (1)	36.4 (16)	36.4 (16)	25.0 (11)	9.1 (3)	21.2 (7)	51.5 (17)	18.2 (6)	1.4 (1)	28.6 (29)	35.7 (25)	34.3 (24)	7.6 (5)	30.3 (20)	42.4 (28)	19.7 (13)
	SARS-CoV-2 detected	4.5 (7)	22.3 (35)	50.3 (79)	22.9 (36)	6.8 (3)	29.5 (13)	45.5 (20)	18.2 (8)	6.1 (2)	21.2 87)	60.6 (20)	12.1 (4)	2.9 (2)	15.7 (11)	41.4 (29)	40.0 (28)	6.1 (4)	28.8 (19)	47.0 (31)	18.2 (12)
	Positive blood culture	4.5 (7)	32.5 (51)	48.4 (76)	14.6 (23)	2.3 (1)	36.4 (16)	45.5 (20)	15.9 (7)	3.0 (1)	9.1 (3)	54.5 (18)	33.3 (11)	2.9 (2)	31.4 (22)	42.9 (30)	22.9 (16)	6.1 (4)	25.8 (17)	48.5 (32)	19.7 (13)

<u>Table S7</u> <u>Absolute and relative frequencies in the analysis of clinical and ancillary criteria in relation to medical specialty.</u>

79.6% (n=35/44) of surgeons considered a postoperative status to be an indication for a CT examination, whereas significantly fewer (50.0%, n=35/70) anesthesiologists agreed (p=0.013). While 81.9 % (n=54/66) of physicians from other medical specialties and 79.6% (n=125/157) of internists classified immunosuppression as a criterion supporting a CT request, only 57.2% (n=40/70) of anesthesiologists agreed (p=0.020). Compared with other medical specialties, significantly more radiologists (96.9%, n=32/33) classified “fever or hypothermia” as a clinical parameter supporting a CT examination in septic patients (p<0.001).

Differences in responses were found for "elevated C-reactive protein (CRP)" related to the physicians' medical specialty (p<0.001). Compared with other medical specialties, radiologists (87.9%, n=29/33) and surgeons (93.2%, n=41/44) showed a strong tendency to consider elevated CRP as a relevant factor for requesting a CT. Significant differences were also seen in responses between medical specialties regarding "leukocytosis or leukopenia" (p=0.002). Even though 67.5% (n=106/157) of internal medicine physicians considered the leukocyte count an argument for a CT scan, this percentage was significantly lower than for surgeons (93.2%, n=41/44) or radiologists (96.9%, n=32/33). In addition, at 63.6% (n=21/33), radiologists tended to agree significantly more often that an elevated IL-6 value supports a CT request than physicians from other medical specialties (p=0.005).

1= strongly disagree; 2= somewhat disagree; 3= somewhat agree; 4= strongly agree

CT = Computed tomography; SOFA = Systemic Organ Failure Assessment; qSOFA = Quick Systemic Organ Failure Assessment; SIRS = Systemic Inflammatory Response Syndrome; RR = Respiratory rate; SBP = Systolic blood pressure; MAP = Mean arterial pressure; PCT = Procalcitonin; CRP = C-reactive protein; IL-6 = Interleukin-6; SARS-CoV-2 = Severe acute respiratory syndrome coronavirus type 2
